# Forskolin-driven conversion of human somatic cells into induced neurons through regulation of the cAMP-CREB1-JNK signaling

**DOI:** 10.7150/thno.92700

**Published:** 2024-02-11

**Authors:** Guodong Wang, Dandan Zhang, Liangshan Qin, Quanhui Liu, Wenkui Tang, Mingxing Liu, Fan Xu, Fen Tang, Leping Cheng, Huiming Mo, Xiang Yuan, Zhiqiang Wang, Ben Huang

**Affiliations:** 1 Guangxi Key Laboratory of Eye Health, Department of Technical Support, The People's Hospital of Guangxi Zhuang Autonomous Region, Guangxi Academy of Medical Sciences, Nanning, 530021, China.; 2 School of Animal Science and Technology, Guangxi University, Nanning, 530005, Guangxi, China.; 3 Key Laboratory of Longevity and Aging-related Diseases of Chinese Ministry of Education, Guangxi-ASEAN Collaborative Innovation Center for Major Disease Prevention and Treatment, and Guangxi Key Laboratory of Regenerative Medicine, Center for Translational Medicine, Guangxi Medical University, Nanning, 530021, China.

**Keywords:** Chemically induced neurons (ciNs), Somatic cell transdifferentiation, Forskolin, CREB1-JNK, Regulatory network

## Abstract

Human somatic cells can be reprogrammed into neuron cell fate through regulation of a single transcription factor or application of small molecule cocktails.

**Methods:** Here, we report that forskolin efficiently induces the conversion of human somatic cells into induced neurons (FiNs).

**Results:** A large population of neuron-like phenotype cells was observed as early as 24-36 h post-induction. There were >90% TUJ1-, >80% MAP2-, and >80% NEUN-positive neurons at 5 days post-induction. Multiple subtypes of neurons were present among TUJ1-positive cells, including >60% cholinergic, >20% glutamatergic, >10% GABAergic, and >5% dopaminergic neurons. FiNs exhibited typical neural electrophysiological activity in vitro and the ability to survive in vitro and in vivo more than 2 months. Mechanistically, forskolin functions in FiN reprogramming by regulating the cAMP-CREB1-JNK signals, which upregulates cAMP-CREB1 expression and downregulates JNK expression.

**Conclusion:** Overall, our studies identify a safer and efficient single-small-molecule-driven reprogramming approach for induced neuron generation and reveal a novel regulatory mechanism of neuronal cell fate acquisition.

## Introduction

Neurons are some of the most important cells in the body and control a wide range of physiological activities 1. Mature neurons naturally lose their proliferation and regeneration abilities, and neuronal damage, especially to brain neurons, can cause severe motor dysfunction 2. However, the regeneration of functional neurons after neuronal injury remains a major challenge 3, 4. Recent studies have demonstrated that somatic cells (fibroblasts, glial cells and astrocytes) can be converted into functional neurons both in vitro and in vivo through regulation of the expression of transcription factors or induction with small molecule cocktails 5-10. Thus far, viral-based expression of transcription factors has been largely used for the conversion of somatic cells into neurons 5,10; however, this approach introduces exogenous genes, limiting its translation into clinical applications 11. In contrast, small molecule cocktails that target signaling pathways, epigenetic modifications, or metabolic processes are also capable of directly reprogramming somatic cells into neuron progenitor cells 12 or neurons 6-9. Compared to transcription factor-based reprogramming, the small molecule-induced reprogramming approach is advantageous because it is nonviral, does not require transcription factors, is cost effective, is easy to alter and standardize, and has a broad range of downstream applications 13. Therefore, small molecule strategies could potentially be translated into clinical therapeutic applications. However, the small molecule cocktails currently used for reprogramming include several small molecules that may cause unpredictable potential side effects, since their induction effects are complicated and the reprogramming mechanisms have still not been elucidated 11. These issues have significantly impeded the further clinical application of small molecules in neuronal regeneration.

Forskolin is a diterpene produced by the roots of the Indian plant Coleus forskohlii 14. The natural small molecule compound forskolin, which has a low molecular weight and easily crosses the cell membrane and internal tissue barrier, has been used for centuries in traditional medicine, and its safety has also been documented in modern medicine 14, 15. Forskolin directly activates the adenylate cyclase enzyme (AC), which generates cAMP from ATP, thus increasing intracellular cAMP levels 16, and is commonly used to reduce body fat 17. Moreover, the increases in intracellular cAMP levels can also increase the expression of PKA/CREB1, which is beneficial for neuronal survival and health because it inhibits apoptosis signaling pathways, such as the JNK signaling pathway 18, 19. Previous reports have shown that forskolin can be used as a small molecule compound to promote the neural differentiation of mesenchymal stem cells 20 and the generation of chemically induced neurons (ciNs) by small molecule cocktails 6, 7; however, forskolin is reportedly not a critical small molecule for the conversion of somatic cells into ciNs 8.

Surprisingly, in the current study, we discovered that forskolin induction alone can highly efficiently reprogram human somatic cells directly into induced neurons (FiNs), including a wide range of neuronal-subtype cells, which has never been described. These FiNs can survive for >2 months in culture and display significant robust neural electrophysiological activity. Injecting these induced neurons into the mouse brain in vivo revealed that these human FiNs can survive for >2 months. Moreover, our findings demonstrate that forskolin participates in the conversion of somatic cells into FiNs by regulating the cAMP-CREB1-JNK signals. The regulatory effects of any single site of this pathway can induce this conversion successfully. Therefore, this study identifies a natural small molecule for neuronal regeneration with a clear regulatory mechanism and may offer a novel strategy for clinical application in the treatment of neurodegenerative disease.

## Results

### Conversion of somatic cells into neuronal cells by forskolin induction

**Figure 1:**
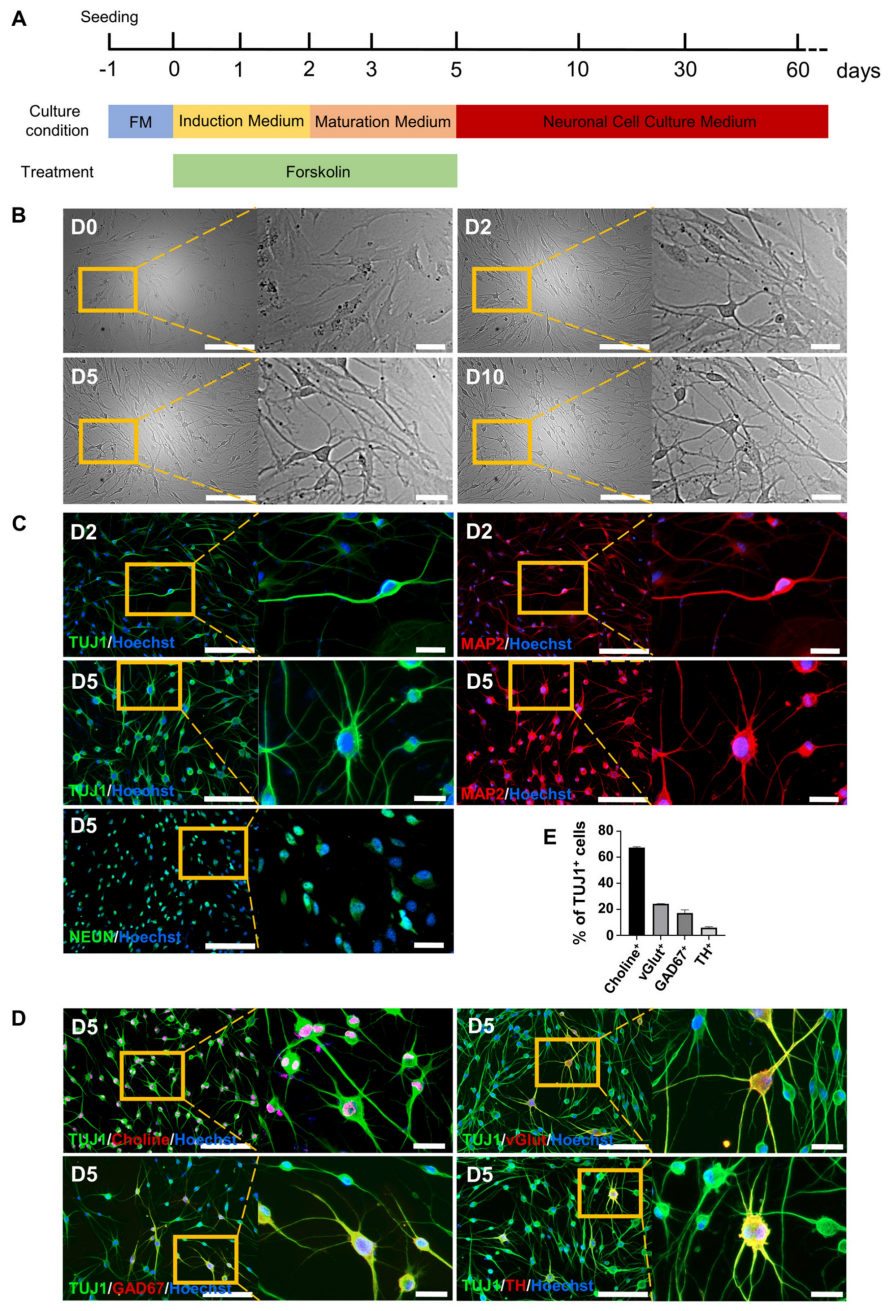
** Conversion of somatic cells into neuronal cells by forskolin induction.** A. Diagram of the forskolin induction process and induced neuron culture. B. Brightfield and regional magnification of cell phenotypes with the same field at days 0, 2, 5, and 10. Scale bars, 200 μm. Magnification scale bars, 40 μm. C. Immunofluorescence staining for the neuronal markers TUJ1 (green), MAP2 (red) and NEUN (green) on days 2 (D2) and 5 (D5). Scale bars, 200 μm. Magnification scale bars, 40 μm. D. Immunofluorescence staining for neuronal subtype markers, choline (red), vGlut (red), GAD67 (red), and TH (red), at D5. TUJ1-positive cells are shown in green. Scale bars, 200 μm. Magnification scale bars, 40 μm. E. Percentages of choline- (cholinergic marker), vGlut- (glutamatergic marker), GAD67- (GABAergic marker), and TH- (dopaminergic marker)-positive cells among TUJ1-positive cells at D5.

**Figure 2:**
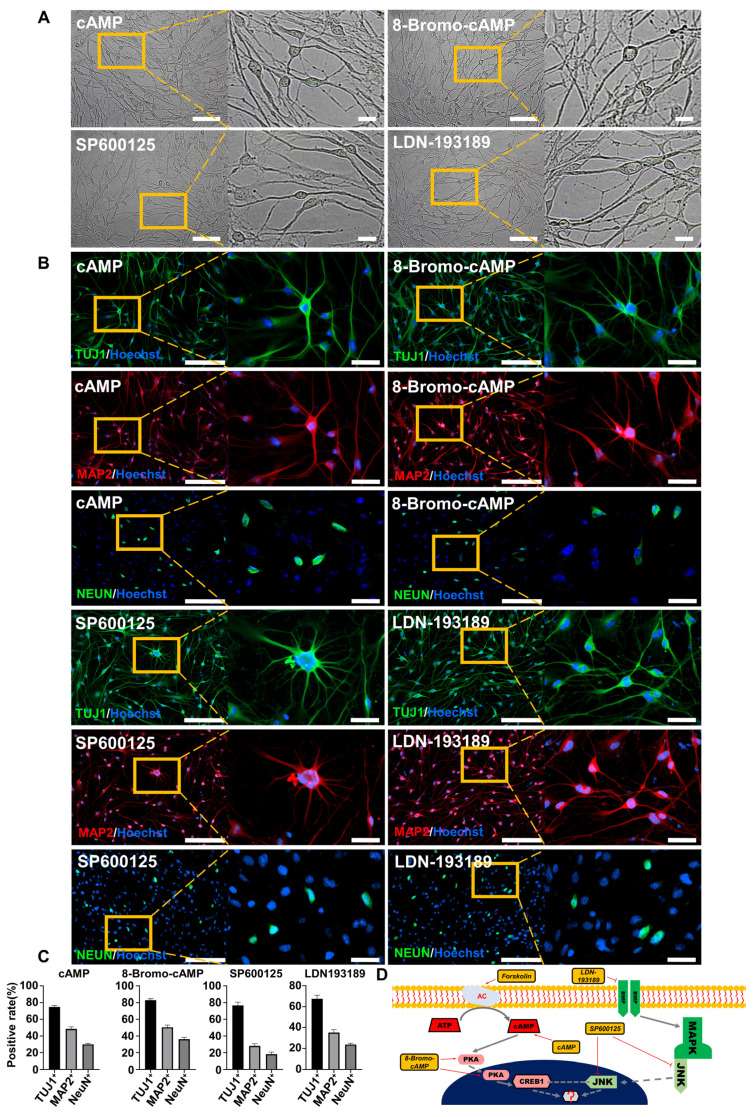
** The cAMP-CREB1-JNK signals determines the cell fate conversion of BJ cells into neuronal cells under forskolin induction.** A. Brightfield and regional magnification of the phenotype of neurons generated by only cAMP, 8-Bromo-cAMP, SP600125 or LDN193189 induction, respectively. Scale bars, 100 μm. Magnification scale bars, 20 μm. B. The neuronal markers TUJ1 (green), MAP2 (red), and NEUN (green) were expressed in neurons generated by only cAMP, 8-Bromo-cAMP, SP600125 or LDN193189 induction, respectively. Scale bars, 200 μm. Magnification scale bars, 40 μm. C. The positive rate of TUJ1, MAP2 and NeuN immunofluorescence of induced neurons generated by cAMP, 8-Bromo-cAMP, SP600125 and LDN193189 induction, respectively. D. Schematic diagram of the hypothetical regulatory pathways and sites of action of small molecules for the conversion of BJ cells into neuronal cells.

Our previous study 21 showed that a small chemical cocktail, BFRTV (B, TTNPB; F, forskolin; R, RepSox; T, tranylcypromine; and V, VPA), could induce fibroblasts to reprogram into mammary epithelial cells derived from the embryonic ectoderm. We then hypothesized that BFRTV might be able to induce the conversion of fibroblasts into other ectoderm-derived cells, such as neurons. Through further small molecule compound (BFRTV) screening, we surprisingly discovered that many BJ cells (human skin fibroblasts) treated with induction medium (IM) (including 10 μM forskolin; F) exhibited a bipolar neuron-like cell morphology as early as 24-36 h post-induction and significantly exhibited this morphology at 2-3 days post-induction (Figure 1A-B, Figure S1A and Supplemental Video). These bipolar neuron-like cells yielded >50% TUJ1-positive cells and >20% MAP2-positive cells at 2 days post-induction (D2). Subsequently, we replaced the induction medium with neuron maturation medium (including 10 μM forskolin). On day 5 (D5), the positive rates of TUJ1 and MAP2 were greater than 90% and 80%, respectively, and >80% of the cells expressed the mature neuronal marker NEUN (Figure 1C and S1B). BJ cells (day 0) stained negative for the neuronal markers TUJ1, MAP2 and NEUN and were used as negative controls. Human induced neuronal cells generated from somatic cells by induction with a small molecule cocktail (VCRFSGY) as described in a previous report 7 stained positively for TUJ1, MAP2 and NEUN and were used as positive controls (Figure 1C and S1C-D). Subsequently, these FiNs survived >2 months in vitro in neuronal cell culture medium without the addition of 10 μM forskolin (Figure 2B, S1A, S2A-C). Meanwhile, the human astrocyte marker antibody GFAP did not significantly stain positive during this period, and BJ cells (D0) expressed the fibroblast marker VIM (Figures S3 A). In addition, the results of quantitative real-time PCR (qRT-PCR) were consistent with the above immunofluorescence (IF) results, showing that the expression of fibroblast marker genes was significantly downregulated, while that of neuronal marker genes was significantly upregulated (Figures S3 B-C). Moreover, at 5 days post-induction (D5), the ratios of cells positive for the neuronal subtype markers choline, vGlut1, GAD67 and TH to TUJ1-positive cells were more than 60%, 20%, 10%, and 5%, respectively (Figure 1D-E). These findings indicate that BJ cells can be rapidly, easily, and efficiently reprogrammed into multiple subtypes of neurons, including cholinergic, glutamatergic, GABAergic, and dopaminergic neurons, by using a single small molecule, forskolin, without the appearance of astrocytes during the whole process. Moreover, human adult somatic cells (human adult skin fibroblasts, HSFs and human adult ovarian granule cells, HGCs) also can be efficiently converted into induced neurons under forskolin induction (Figure S4). Therefore, forskolin is able to efficiently induce the conversion of human somatic cells into neurons.

### The cAMP-CREB1-JNK signals determines the cell fate conversion of BJ cells into neurons under forskolin induction

To further investigate the regulatory mechanism of FiNs in the reprogramming process, we screened small molecule compounds that act on forskolin induction-related signaling pathways. Interestingly, the results showed that the addition of cAMP (1 mM), 8-bromo-cAMP (PKA/CREB1 activator; 50 μM), SP600125 (JNK inhibitor; 10 μM) or LDN193189 (BMP/ALK2,3 inhibitor; 2.5 μM) within induction medium (no forskolin) could also reprogram BJ cells into neurons (Figure 2A), as determined by positive staining for the specific neuronal markers TUJ1, MAP2, and NEUN (Figure 2B-C). Therefore, based on known signaling pathway information, we speculate that the cAMP-PKA/CREB1-JNK signals may play a decisive role in FiN reprogramming from somatic cells (Figure 2D).

**Figure 3:**
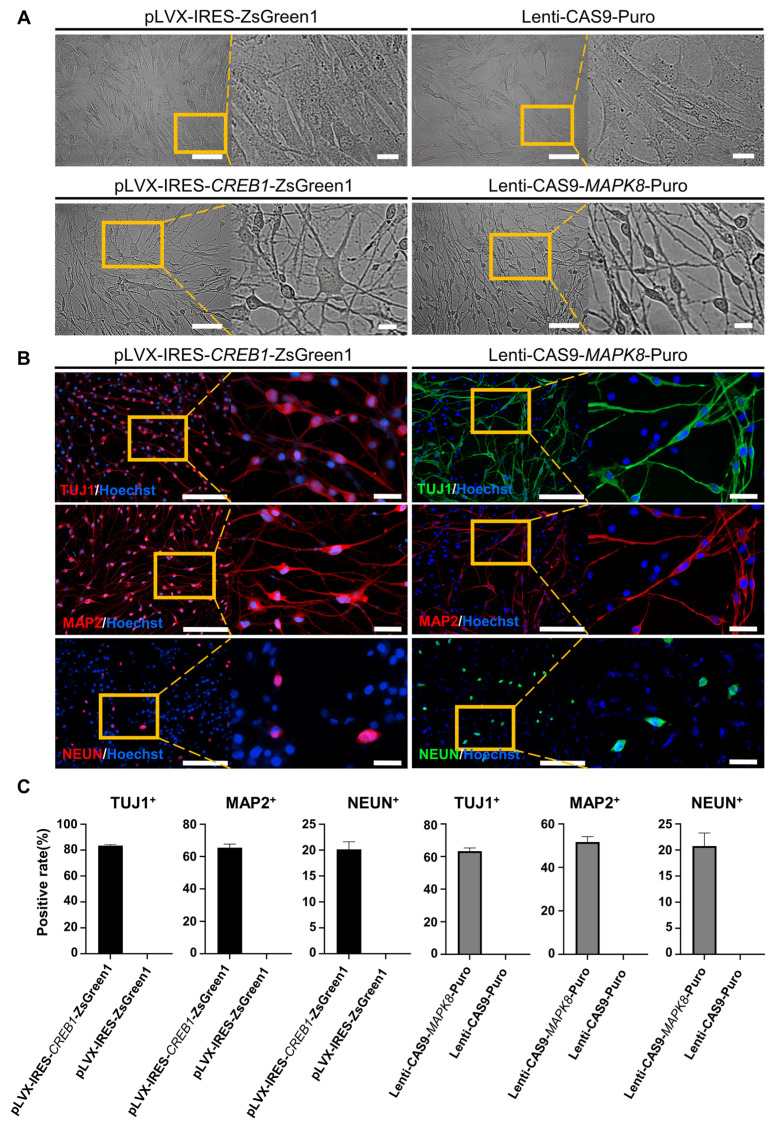
** CREB1 and JNK (MAPK8) are key regulatory genes for the conversion of BJ cells into FiNs.** Brightfield and regional magnification of induced neurons generated by *CREB1* overexpression with pLVX-IRES-*CREB1*-ZsGreen1 or *JNK* (*MAPK8*) downregulation with Lenti-CAS9-*MAPK8*-Puro. BJ cells transfected with pLVX-IRES-ZsGreen1 (empty vector) or Lenti-CAS9-Puro (empty vector) acted as negative controls. Fibroblast medium was replaced with neuronal IM without the addition of forskolin at two days post-transfection. Subsequently, cells were cultured in neuronal MM (without the addition of forskolin) and NM as described at Figure 1A. Scale bars, 100 μm. Magnification scale bars, 20 μm. Induced neurons generated by *CREB1* overexpression or *JNK* (*MAPK8*) downregulation expressed the neuronal markers TUJ1, MAP2, and NEUN. Scale bars, 200 μm. Magnification scale bars, 40 μm. C. The TUJ1-, MAP2-, and NEUN-positive rates in cells induced with *CREB1* overexpression or *JNK* (*MAPK8*) downregulation.

To demonstrate the regulatory pathway and the key genes that regulate this reprogramming process, we further conducted gene overexpression and knockdown experiments to confirm the regulatory effects of *CREB1* and *JNK (MAPK8)*. The results showed that *CREB1* overexpression with pLVX-IRES-*CREB1*-ZsGreen1 or *JNK (MAPK8)* downregulation with Lenti-CAS9-*MAPK8*-Puro could reprogram BJ cells into neurons, as determined by the neuronal cellular phenotype and positive IF staining of neuronal markers (TUJ1, MAP2 and NEUN) (Figure 3A-B and Figure S5 A-C). In contrast, *JNK* overexpression with pLVX-IRES-*MAPK8*-ZsGreen1 or *CREB1* downregulation with Lenti-CAS9-*CREB1*-Puro significantly decreased the rates of TUJ1- and MAP2-positive neurons after SP600125 or forskolin induction, respectively (Figures S5 D-E). Therefore, our findings suggest that *CREB1* and *JNK (MAPK8)* are critical regulatory genes for FiN reprogramming and demonstrate that forskolin functions in this reprogramming through the regulation of the cAMP-CREB1-JNK signals.

### FiNs show typical neural electrophysiological properties

**Figure 4:**
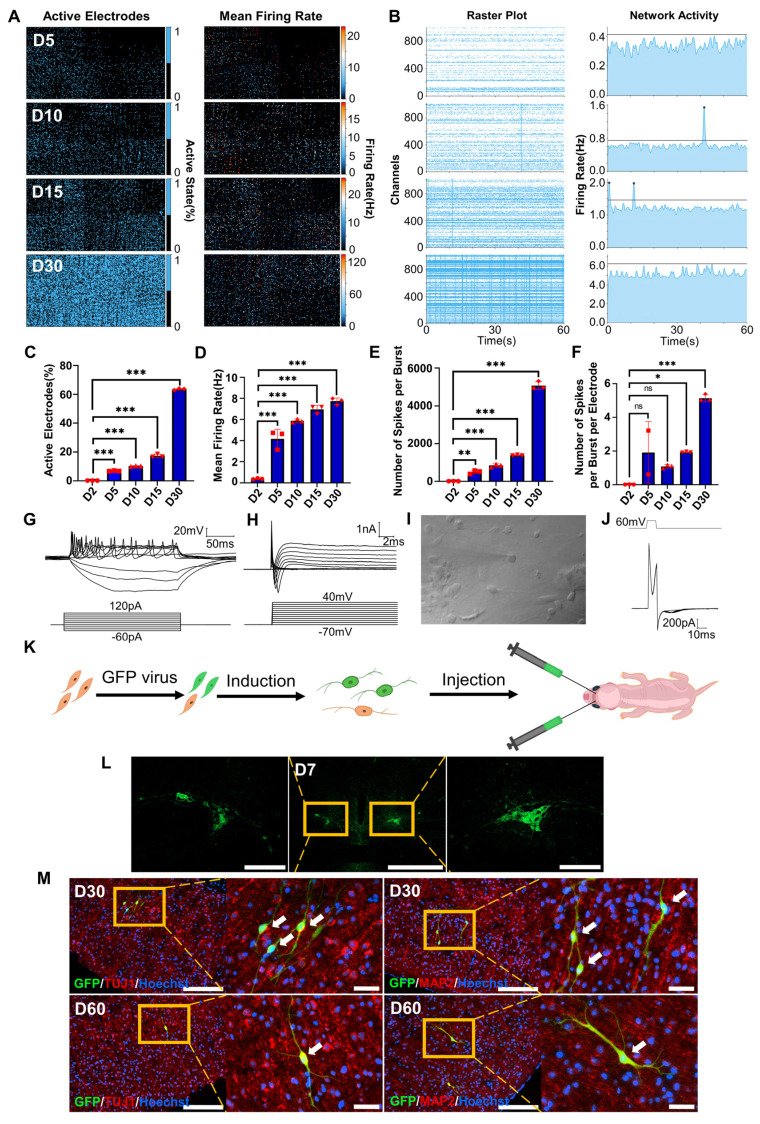
** Electrophysiological properties of FiNs and transplanted FiN survival in mouse brains.** A. HD-MEA electrical images showing 2D spatial distribution maps of the active electrodes (%) (left) and mean firing rate (Hz) (right) on days 5, 10, 15 and 30. B. HD-MEA chips detected the electrical signals of cells in each channel (left) and the network activity (firing rate (Hz)) (right) with a cycle of 60 seconds on days 5, 10, 15 and 30. C-F. Active electrode rates (C), mean firing rate (Hz), (D) number of spikes per burst (E) and number of spikes per burst per electrode (F) of cells detected in HD-MEA chips on days 2, 5, 10, 15, and 30 (mean ± SEM, n=3 biological replicates, *p < 0.05, **p < 0.01, ***p < 0.001, one-way ANOVA). G. Current-clamp recordings of FiNs showing a representative train of action potentials (top panel). Step currents were injected from -60 pA to 120 pA (bottom panel). H. Large currents of voltage-dependent sodium and potassium channels. I. Representative trace of spontaneous postsynaptic currents in FiNs. J. Synaptic currents evoked by avoltage step (60mV, 1ms) in the voltage-clamp mode. K. Schematic diagram of the bilateral intracerebral injection of FiNs. L. On day 7 after FiN injection (D7), cryosectioning showed green fluorescence (GFP) at the injection site. Scale bars, 1000 μm. Magnification scale bars, 200 μm. (n= 3 injected mice for analysis). M. On days 30 (D30) and 60 (D60) after FiN injection, cryosection IF showed FiNs with GFP labels that survived and expressed the neuronal markers TUJ1 (red) and MAP2 (red) in the mouse brain. Scale bars, 200 μm. Magnification scale bars, 40 μm. White arrows indicate transplanted GFP-FiNs. (n= 6 injected mice of each timepoint for analysis).

To investigate the electrophysiological properties of FiNs, we used high-density microelectrode arrays (HD-MEAs) to detect the cells induced by forskolin for 0 days, 2 days, 5 days, 10 days, 15 days and 30 days. During the forskolin induction process, the percentage of active electrodes on the HD-MEA chip gradually expanded from approximately 0.25% (D2, 2 days post-induction) to approximately 63% (D30, 30 days post-induction), and the mean firing rate (Hz) also gradually increased (Figure 4A, C-D and Figure S6 A-B). Neuronal networks are often characterized by synchronized activity (bursts) resulting from recurrent synaptic connections that form as the neuronal network matures. Testing of the different induction time groups mentioned above and raster plotting revealed that network activity, the number of spikes per burst and the number of spikes per burst per electrode increased significantly with increasing induction time (Figure 4B, E-F). Moreover, we conducted whole-cell recording of FiNs and found that at 15 days post induction, FiNs were able to generate an action potentials (APs) in response to current clamp mode injection of depolarized step currents, and possess fast-decay spontaneous excitatory postsynaptic currents (sEPSCs). Therefore, these results are consistent with that of HD-MEAs method (Figure 4G-J). Moreover, the actual neurotransmitters (dopamine and GABA) were detected in the cell supernatant of induced neurons at 5 days post forskolin induction (Figure S6 C). Collectively, these findings suggest that FiNs possess typical neural electrophysiological activity.

### Survival of transplanted FiNs in mouse brains

To demonstrate whether transplanted FiNs could survive for long term in vivo, we conducted in vivo transplantation experiments. BJ cells were transfected with GFP-labeled lentivirus and then induced with forskolin for 2 d to generate GFP-FiNs. The GFP-FiNs-2d were trypsinized and injected bilaterally into the lateral ventricles of postnatal day 1 mice (Figure 4K). At 7, 30 and 60 days post-injection (DPI), mice were sacrificed for cryosectioning, and obvious green fluorescence was observed at the injection site at 7 DPI (Figure 4L). Subsequently, some transplanted cells with GFP in cryosections at 30 and 60 DPI were stained red by IF, which indicated that they expressed the neuronal markers TUJ1 and MAP2 (Figure 4M). These findings indicate that FiNs can survive >2 months when transplanted into mouse brains. Moreover, a few GFP-cells resembling neurons were not positive for TUJ1, which may suggest that these cells were dying-induced neurons, incompletely reprogrammed neurons or GFP-positive fibroblasts. However, additional experiments need to be conducted to clarify this point.

### Single-cell sequencing and mRNA-seq analyses demonstrate the cell fate conversion of BJ cells into FiNs

**Figure 5:**
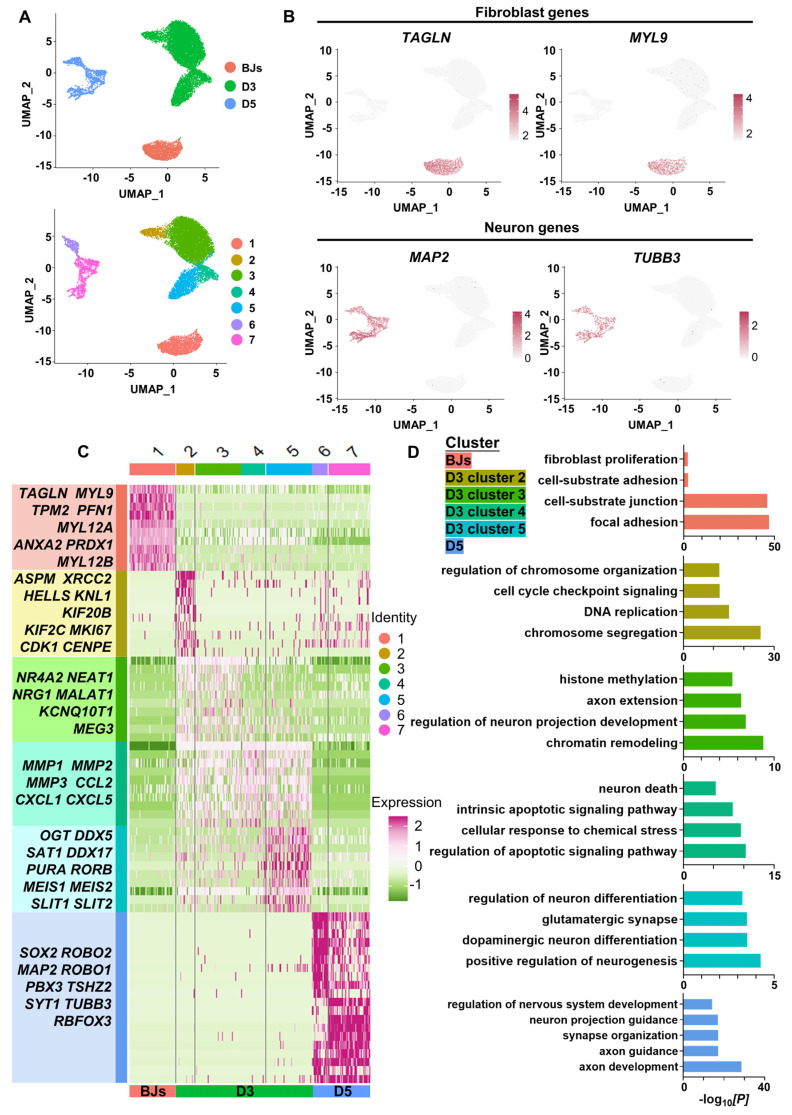
** ScRNA-seq analyses demonstrate the conversion of BJ cells into FiNs.** A. Uniform manifold approximation and projection (UMAP) analysis of the BJ cells (D0), cells at 3 days post-induction (D3) samples and cells at 5 days post-induction (D5) samples (left). The UMAP plots display the induced cells collected on D0 (BJ cells), D3 and D5 that were clustered into 7 clusters (right). B. UMAP feature plots of the expression of the fibroblast marker genes* TAGLN* and *MYL9* and the neuron marker genes *MAP2* and *TUBB3.* C. Heatmap showing the differentially expressed genes (DEGs) cataloged in each cluster. D. GO analysis showing the enriched terms in each cluster.

To dissect the molecular events during FiN reprogramming, we performed single-cell RNA (scRNA)-seq to investigate the transcriptomes of individual cells collected at three time points along the reprogramming path: initiating BJ cells (BJs), cells at 3 days post-induction (D3) and cells at 5 days post-induction (D5). Using the unsupervised dimensional reduction and visualization method of uniform manifold approximation and projection (UMAP) plotting, we clustered cells from all stages into seven cell clusters (Figure 5A). Based on the marker genes for each cluster and the stages of the cells collected, the cells of cluster 1 expressing the fibroblast markers *TAGLN* and *MYL9* were classified as initiating BJ cells, while the cells of clusters 6 and 7 within the sample at 5 days post-induction (D5) exhibited some neuron specific markers such as *MAP2* and *TUBB3* (Figure 5B).

Moreover, based on the marker genes for each cluster, we first determined that cells of clusters 6 and 7 at 5 days post-induction (D5) were cells that had been successfully reprogrammed into induced neurons, which expressed a number of neuron-specific markers, including *ASCL1, NEUROG2, NEUROD1, RBFOX3*(*NEUN*)*, MAP2, TUBB3*(*TUJ1*), etc., and the enrichment of neuronal-related GO terms (Figure 5 C-E). Some neural development and functional synapse related genes, such as the *MEIS2*, *DDX5*, *SAT1*, *PURA* and* RORB,* were expressed specifically in the cells of cluster 5 at 3 days post-induction (D3) (Figure 5C-D, Figure S7A), which indicated that the cells of cluster 5 more likely follow-up development into neurons. Second, we found that the cells of cluster 2 at 3 days post-induction dominantly expressed cell cycle-related genes (*MKI67*, *CDK1*, *CENPF,* etc.), which were enriched with cell cycle-related GO terms, while fibroblast-specific genes were downregulated (Figure 5C-D, Figure S7A). Interestingly, genes associated with early embryonic neurodevelopment were upregulated at the same time (*ASPM*, *KIF20B*, *KNL1,* etc.) (Figure 5C). These findings indicate that these cells of cluster 2 may have been in a preparatory state of neuronal lineage commitment. Third, compared with the cells of cluster 2, the cells of cluster 3 showed obvious downregulation of cell cycle-related genes followed by upregulation of a panel of genes involved in processes of neural differentiation and regeneration, such as *MALAT1* (regulation of synaptogenesis and neurogenesis), *NEAT1* (regulation of neuronal excitability), and *NRG1* (the major synaptogenic protein) (Figure 5C). This may suggest that the cells of cluster 3 had already entered the neuronal lineage and were in an intermediate state of FiN reprogramming. Fourth, the cells of cluster 4 were enriched with many terms related to cell death and apoptosis, and neural-lineage genes were not significantly expressed, which did not seem to indicate successful reprogramming (Figure 5C-D). These findings indicated that four different cellular states may have been captured in the reprogramming route from BJ cells to FiNs. The above findings from the scRNA-seq analyses may indicate that forskolin can efficiently reprogram BJ cells into neuronal cells after forskolin induction.

**Figure 6:**
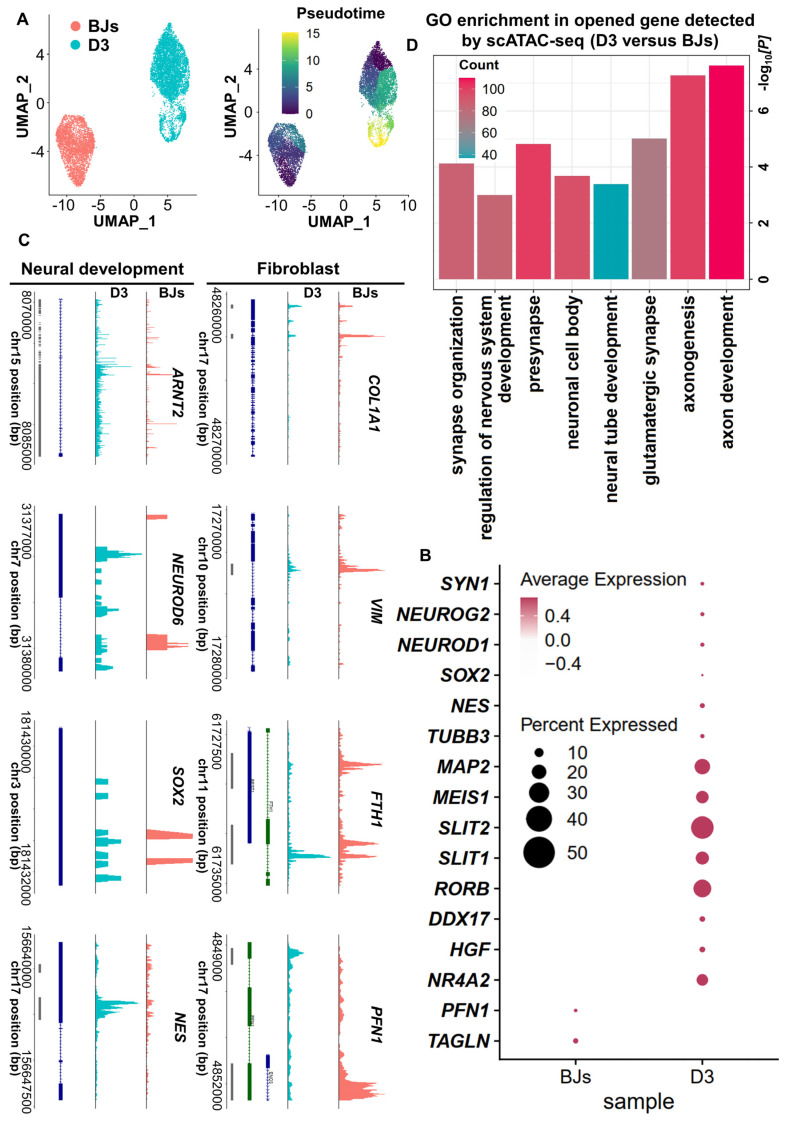
** ScATAC-seq analyses demonstrate the conversion of BJ cells into FiNs.** A. scATAC-seq analysis of BJ cells and D3 cells. The UMAP plots show that the induced cells collected on days 0 (BJ) and 3 (D3) (left). Color-coding was performed pseudotime (right). The UMAP overlay of pseudotime implies developmental progression. B. Dotplot showing the neural development- and neuron marker gene open access in the D3 cells sample. C. Genome tracks showing scATAC accessibility at the neural development gene locus are highlighted in the D3 cells sample, and the fibroblast gene locus is highlighted in the BJ sample. D. GO analyses of significantly opened genes of the D3 cells sample versus the BJ cells sample. The P value was determined by a one-sided hypergeometric test without adjustments.

Based on the above findings from scRNA-seq analysis, the cell fate was changed significantly at 3 days post-induction. It may indicate that 3 days post-induction is the critical timepoint for FiN cell fate decision. In order to further clarify this point, we used a single-cell assay for transposase-accessible chromatin sequencing (scATAC-seq) to analyze BJs and 3 days post-induction cell samples (D0 and D3 cells), and the pseudo-time analysis showed that the D3 sample gradually transitioned from the fibroblast state (Figure 6A). The results revealed an open chromatin state of the cells at 3 days post-induction with increased accessibility at certain gene loci related to neural development and decreased accessibility at certain gene loci related to fibroblasts. However, the cells in BJ cells sample, showed chromatin accessibility profiles opposite those of the cells in D3 sample (Figure 6B-C). Gene Ontology (GO) analysis showed that the genes that were significantly activated at 3 days post-induction were enriched for the neural development related terms (Figure 6D). These findings are consistent with those of the above scRNA-seq analysis, and further demonstrated that some induced cells at 3 days post-induction had entered the neural cell fate commitment and subsequently had chances to develop into neuron cell fate at 5 days post-induction.

Finally, in parallel to the scRNA/ATAC-seq analysis, we collected samples at D0, D2 and D5 to measure the global gene expression profiles by mRNA sequencing (mRNA-seq). The differentially expressed genes (DEGs) were grouped according to their expression patterns during the induction process. The genes in the upregulated group were enriched with GO terms related to neurogenesis, while the genes in the downregulated group were enriched with terms related to fibroblasts (Figure S7 B). Moreover, Kyoto Encyclopedia of Genes and Genomes (KEGG) enrichment analysis showed that neuronal subtype terms were enriched for the D5 samples (Figure S7 C) and that neuronal subtype-related genes were upregulated gradually during the induction process (Figure S8 A-E). These findings from mRNA-seq analysis indicated that fibroblast-related genes were downregulated and neuronal-related genes were upregulated during the induction process and that BJ cells ultimately achieved neuronal cell fate. These findings were consistent with the findings of scRNA/ATAC-seq analysis. Overall, the multiomics sequencing analysis demonstrated that the conversion of forskolin-induced neurons (FiNs) from somatic cells was an authentic phenomenon.

### ScRNA-seq reveals a successful FiN reprogramming path

**Figure 7:**
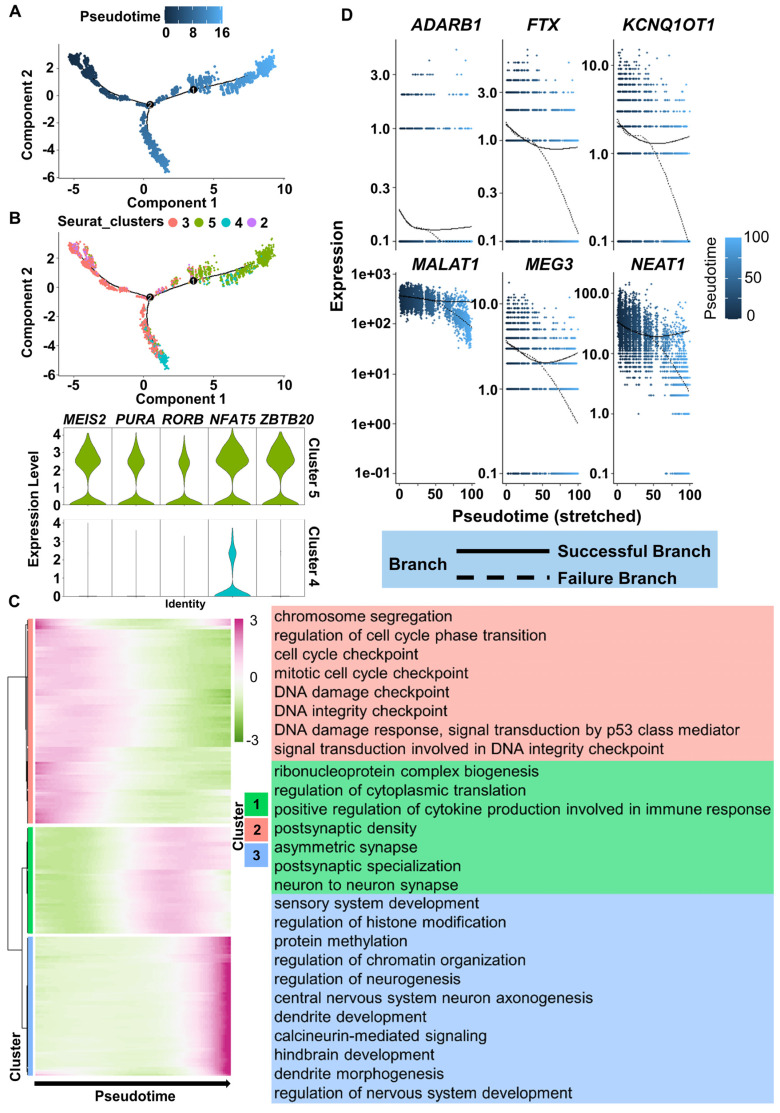
** ScRNA-seq analyses reveal the successful reprogramming events of FiN reprogramming.** A. Monocle-generated pseudotime trajectory of a subsampled population of cells (n = 2000) from each cluster in the D3 cells sample of scRNA-seq data. Pseudotime is shown colored in a gradient from dark to light blue. B. Trajectory reconstruction of three branches in scRNA-seq data: the pre-branch (before bifurcation), the successful branch, and the failed branch (after bifurcation). Cluster 3 and cluster 8 indicate cells at the termini of the successful of neural lineage commitment and failed branches, respectively. Violin plot of scRNA-seq data displaying the expression of representative neural development related genes in the successful and failed branches of neural lineage commitment, respectively. C. Heatmap showing the expression patterns of key dynamically expressed genes along the reprogramming pseudotime (left). The enriched GO terms for each gene set cluster in the heatmap (right). D. Expression pattern scatter plot showing the expression levels and changes/branches in neural development genes that affected successful reprogramming. Solid lines represent successful branches, and dotted lines represent failed branches.

To more precisely understand the FiN reprogramming path, we used Monocle 2 to perform pseudotime ordering analysis to investigate the scRNA-seq data from the cells of the 3 days post-induction samples (cells in clusters 2, 3, 4 and 5). Pseudotime ordering showed that reprogramming was a continuous process that progressed from cluster 2 (pre-branch) through cluster 3 to cluster 5 (neural lineage commitment successful branch) or cluster 4 (failed branch) (Figure 7A-B). The induced cells in cluster 5 significantly expressed neural development related genes, such as the *MEIS2*, *RORB*, *PURA*, *NFAT5* and *ZBTB20*, while these genes were not significantly expressed in the cells of cluster 4 (Figure 7B). Therefore, the above findings indicate that the significant expression of these genes may guarantee successful neuron cell fate commitment.

Moreover, we clustered DEGs into three gene sets based on their expression dynamics along the pseudotime sequence (Figure 7C). The results of GO term enrichment analysis of each gene cluster showed that induced cells in the preparatory state (enriched with cell cycle-related terms, Figure 7C top, red) needed to go through a transient intermediate state (Figure 7C middle, green) to successfully achieve a stable neural lineage fate (enriched with neural development terms, Figure 7C bottom, blue). During the intermediate state, the expression of genes related to neuronal damage repair-related genes, such as *MALAT1*,* MEG3*, *NEAT1*, *KCNQ1OT1*, *FTX*, and the essential for neuronal function gene *ADARB1* were also actively expressed in successful branches of neural lineage commitment rather than in failed branches (Figure 7D). These findings indicate that successful neuron cell fate commitment requires the orderly participation of multiple types of activated regulatory networks to ultimately achieve a neuron identity.

### Regulatory network of FiN reprogramming

The above results demonstrated that the cAMP-CREB1-JNK pathway determines the cell fate conversion of BJ cells into neurons under forskolin induction and that *CREB1* and *JNK (MAPK8)* are critical regulatory genes. Furthermore, the results of scRNA-seq analysis showed that *CREB1* was upregulated after forskolin induction (Figure S9 A). Moreover, scATAC-seq analysis showed that the binding motif of *CREB1* was significantly enriched in the cells in the day 3 (D3) cells sample (Figure S9 B). Interestingly, mRNA-seq analysis showed that a group of genes had transient upregulation in the D2 cells sample, and GO enrichment analysis revealed that the corresponding terms were related to the MAPK/JNK cascade (Figure S9 C). Therefore, these findings further confirm that *CREB1* and *JNK (MAPK8)* are critical regulatory genes in FiN reprogramming, especially at the initial stage.

**Figure 8:**
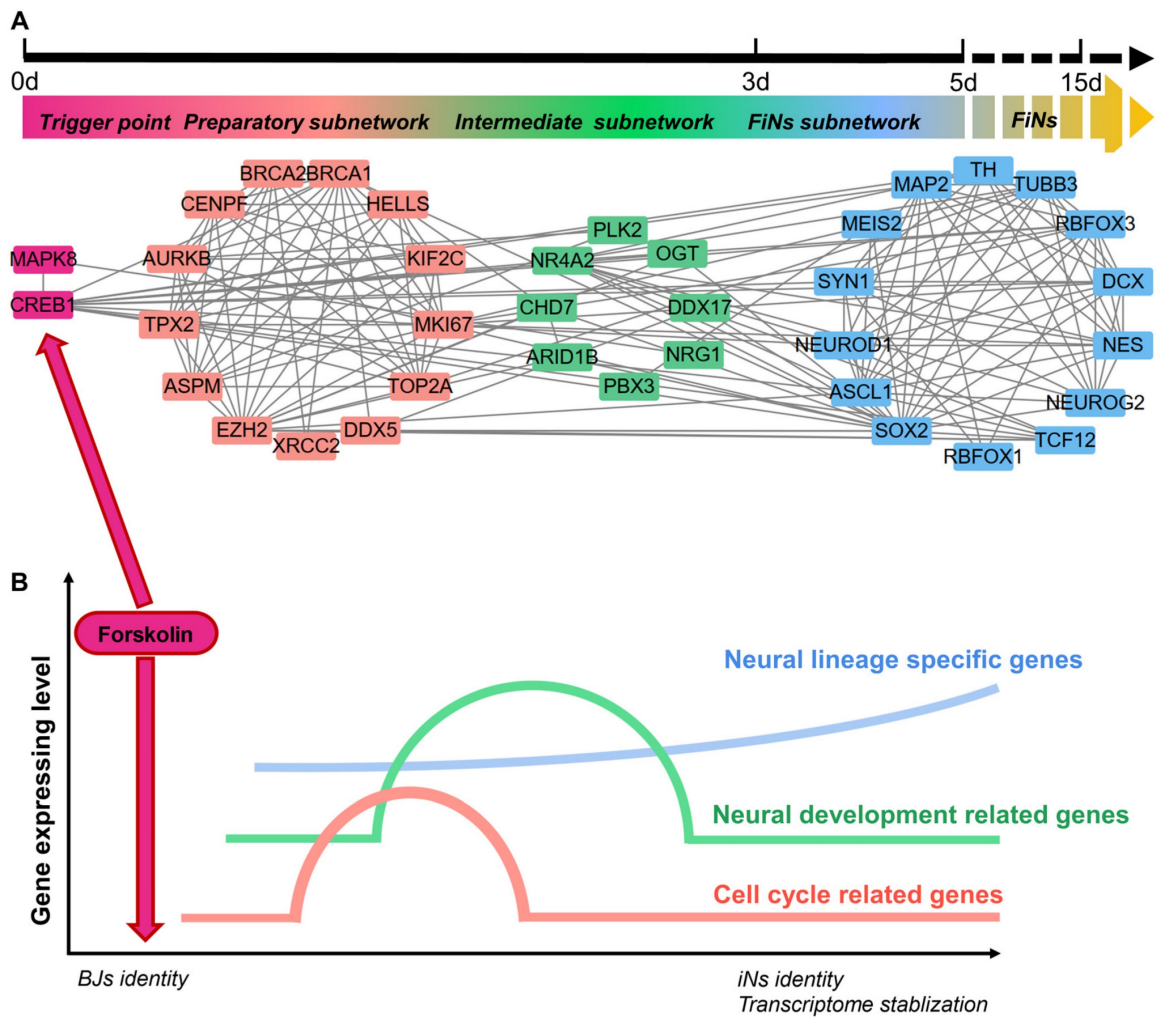
** ScRNA-seq analyses reveal the regulatory network of FiN reprogramming.** A-B. Gene correlation network including triggers, the preparatory subnetwork, the intermediate subnetwork and the FiN subnetwork (E) and (F) a regulatory model summarizing the progression of reprogramming induced by forskolin.

Most importantly, scRNA-seq analysis also revealed the regulatory network of this reprogramming mediated by *CREB1* and *JNK (MAPK8)*. Based on pairwise correlation of the gene expression data, we constructed a regulatory network during progressive cell fate transitions from the trigger (the regulatory effects of *CREB1* or *JNK* induced by forskolin) to FiNs (Figure 8A). In detail, three transcriptional regulatory subnetworks (the preparatory, intermediate and FiN subnetworks) were revealed chronologically. The preparatory-state subnetwork was connected with the trigger genes (*CREB1* or *JNK*) and the transient upregulation of cell cycle- and neural development-associated genes was the result of the regulatory effects of the trigger genes. Moreover, the sequential switching of transcriptional circuits highlighted the intermediate subnetwork as the bridge linking the preparatory state to the FiN state. In fact, the continuously expressed intermediate-state genes may guarantee that the neural lineage-specific genes were expressed until the end of reprogramming to successfully stabilize the neuron cell fate.

These findings indicate that the reprogramming of BJ cells to FiNs requires several transcriptional waves. First, under the regulatory effects of *CREB1* or* JNK* as triggers, cell cycle- and neural development-related genes are transiently upregulated in the preparatory state. Subsequently, the reprogrammed cells enter an intermediate state characterized by significant expression of neural development genes. Finally, neuron identity is further strengthened and stabilized by the continuous expression of these neural lineage-specific genes (Figure 8B).

## Discussion

The conversion of human somatic cells into neuron cell fate via induction with small molecule cocktails has been reported previously 6, 7. However, there are no reports that a single small molecule can induce this conversion. Here, we report a natural small molecule compound, forskolin, which has been safely used in many applications to cure human diseases and maintain human health 14, and can efficiently induce the conversion of human fibroblasts into functional neuronal cells. Forskolin has been commonly used in small molecule cocktail-mediated induction to promote the conversion of somatic cells into neurons but has not been shown to be able to induce this conversion 7, 8.

In our studies, we established a highly efficient induction platform for the conversion of human somatic cells into neuronal cells by using the single small molecule forskolin. This induction platform allowed somatic cells to achieve neuron cell fate rapidly. These cell types were indicated by a large amount of neuron specifical markers-positive cells with typical neural electrophysiological activity, including cells in several neuronal subtypes that survived for >2 months in vitro and vivo. The induction speed and efficiency for the conversion of neuron cell fate form somatic cells were surprisingly faster and higher, respectively, than those of any other neurons reportedly induced from somatic cells. Moreover, this induction method can generate more neuronal subtype cells than any previously reported method 6-9. It may provide huge potential for somatic cells to achieve a wide range of neuron subtype cell fates for the therapy of various types of neuron degeneration diseases in vitro and in vivo. Notably, compared to the reported small molecule cocktail induction approach, our single-small-molecule induction approach may avoid many potential side effects that can arise from different small molecules or their combined effects 11. Such side effects could significantly affect further clinical applications. Moreover, a single small molecule can easily cross the blood-brain barrier and reach the defective tissue to induce neuronal regeneration in vivo; however, it is difficult to guarantee that all compounds of small molecule cocktails can reach the defective tissue in vivo in the proper ratio, which may significantly impact their induction effects. Furthermore, forskolin may be able to be combined with nanobioengineering techniques to establish a strategy for precise induction of in situ neuronal regeneration 22. Overall, these findings may offer a safer, faster and more efficient approach for the generation of neurons chemically induced from somatic cells in vitro and in vivo. This approach can be used for the treatment of neurodegenerative diseases in a wide range of clinical applications.

Surprisingly, according to the signaling pathway related to forskolin's induction effects, we also discovered that other single small molecules, cAMP, 8-bromo-cAMP (PKA/CREB1 activator), SP600125 (JNK inhibitor) and LDN193189 (BMP/ALK2,3 inhibitor), can induce the conversion of human somatic cells into neuronal cells. Moreover, by conducting gene overexpression and downregulation experiments for *CREB1* and *JNK (MAPK8)*, we demonstrated that the cAMP-PKA/CREB1-JNK pathway mediates this reprogramming. Mechanistically, forskolin increases intracellular cAMP levels, subsequently upregulating PKA/CREB1 expression, downregulating JNK expression and activating three transcriptional regulatory networks (the preparatory, intermediate and FiN networks), which induces human somatic cells to reprogram into neurons. Interestingly, the regulatory effects of any single member of the cAMP-PKA/CREB1-JNK pathway can induce this conversion successfully. Therefore, we uncovered a novel regulatory pathway that mediates neuronal cell fate acquisition by somatic cells. Previous reports have indicated that the cAMP-PKA/CREB1 signaling pathway is important for regulating neuronal development 23, differentiation and survival. Activation of this pathway promotes neuronal survival and functional maintenance and repair 24, 25. Moreover, JNK signaling, which is best known for its involvement in propagating proapoptotic signaling, plays a role in neuronal death, and there is evidence that this pathway may operate in various central nervous system (CNS) disease states 26. A previous report indicated that JNK inhibition may aid in the treatment of neurodegenerative diseases 27 by reducing neuronal apoptosis and that activation of the cAMP-PKA/CREB1 signaling pathway can suppress JNK activation and antagonize apoptosis 28, 29. Therefore, in addition to the regulatory effects of the cAMP-PKA/CREB-JNK signaling pathway on neuronal differentiation and survival, we surprisingly discovered a novel regulatory effect of this pathway on the induction of neuronal regeneration from somatic cells. These findings reveal a novel regulatory mechanism for achieving neuronal cell fate and offer a promising therapeutic strategy for neurodegenerative diseases involving the induction of neurons from in situ somatic cells.

Furthermore, we reconstructed the forskolin-induced reprogramming pathway via multiomics sequencing analysis. The results indicated that forskolin-induced somatic cells were directly reprogrammed into neuronal cells. This is consistent with previous reports on chemically induced neuronal cells derived from somatic cells 6, 7. However, we revealed a novel FiN reprogramming pathway by forskolin induction. In brief, at the early state of forskolin induction, under the regulatory effects of *CREB1* upregulation and *JNK* downregulation, the activation of the cell cycle- (*CENPF, MKI67*) 30, 31 and embryonic neurodevelopment-associated genes (*ASPM* and* KIF20B*) 32, 33 may be able to remodel the cell cycle and prepare/create an appropriate environment for neurogenesis. Therefore, from this point of view, our findings are consistent with the previously reported findings that claimed that cell cycle remodeling may be the key point for the initial phase of somatic cell reprogramming 34. During this cell cycle remodeling process triggered by the regulatory effects of *CREB1* and *JNK*, environmental signaling for neurogenesis could be released. With the continuation of induction, the existence of an intermediate state allows the activation of neural development and damage repaired-related genes (*NEAT1, MALAT1, ADARB1, NRG1, ZBTB20, PURA*) 35-40. The successful and continuous expression of the above intermediate state-related genes may provide the basis for successful FiNs reprogramming. Subsequently, the reprogrammed cells enter a state of stable expression of neural lineage-specific genes (*OGT, MEIS2, MAP2* and* TUBB3*) 41-43 to achieve successful FiNs reprogramming (Figure S7 D). This is the first study to clearly describe the reprogramming path and regulatory mechanism by which somatic cells achieve a neuronal cell fate under chemical induction.

Overall, we established a robust, highly efficient, and novel method for chemically inducing the conversion of human somatic cells into neuron cell fate via a single small molecule with a clear regulatory mechanism. Moreover, we revealed that any single small molecule that can upregulate cAMP/CREB1 and/or downregulate JNK signaling is capable of inducing the conversion of neuron cell fate from somatic cells. Therefore, our findings offer insights into the mechanism of neuronal cell fate achievement and identify a potentially powerful and clinically feasible strategy to treat neurodegenerative diseases by replacing lost neurons. Prospectively, application of the small molecule forskolin may be a potential approach for in situ neuronal regeneration for therapeutic purposes.

## Method Details

### Cell culture

BJ cells (human skin fibroblasts) were purchased from the American Type Culture Collection (ATCC) (CRL-2522). This cell type was cultured in DMEM (Gibco, Cat. #11965-092) supplemented with 10% fetal bovine serum (FBS) (fibroblast medium; FM) (WISENT, Cat. #086-150). The cells were washed three times with phosphate-buffered saline (PBS) (Beyotime, Cat. #C0221A) and digested with trypsin (Gibco, Cat. #25200-072) for 1 min at room temperature. The trypsin digestion was stopped with the same volume of DMEM+10% FBS. Then, the cells were split 1:3 into new dishes.

### Generation and culture of induced neurons derived from somatic cells

BJ cells were seeded on poly-D-lysine (PDL) (Gibco, Cat. #A38904-01)-coated dishes and cultured with DMEM+10% FBS to a cell density of 80%. The medium was then replaced with neuronal induction medium (IM). On day 2 (D2), the IM was replaced with neuronal maturation medium (MM), and this medium was replaced every 2 days. On D5, the 10 μM forskolin was removed, and the medium was replaced with neuronal cell culture medium (NM), which was replaced every 2 days.

Neuronal IM: DMEM/F12 (Life Technologies, 11330-032), Neurobasal (Life Technologies, 21103-049) (1:1), 0.5% N2 (Invitrogen, 17502048), 1% B27 (Invitrogen, 17504044), 20% KnockOut™Serum Replacement (Gibco, 10828-028), 10 μM forskolin, 100 μM cAMP, 20 ng/ml bFGF and penicillin/streptomycin.

cAMP IM:DMEM/F12 (Life Technologies, 11330-032), Neurobasal (Life Technologies, 21103-049) (1:1), 0.5% N2 (Invitrogen, 17502048), 1% B27 (Invitrogen, 17504044), 20% KnockOut™Serum Replacement (Gibco, 10828-028), 1100 μM cAMP, 20 ng/ml bFGF and penicillin/streptomycin.

8-Bromo-cAMP IM: DMEM/F12 (Life Technologies, 11330-032), Neurobasal (Life Technologies, 21103-049) (1:1), 0.5% N2 (Invitrogen, 17502048), 1% B27 (Invitrogen, 17504044), 20% KnockOut™Serum Replacement (Gibco, 10828-028), 50 μM 8-Bromo-cAMP, 100 μM cAMP, 20 ng/ml bFGF and penicillin/streptomycin.

SP600125 IM: DMEM/F12 (Life Technologies, 11330-032), Neurobasal (Life Technologies, 21103-049) (1:1), 0.5% N2 (Invitrogen, 17502048), 1% B27 (Invitrogen, 17504044), 20% KnockOut™Serum Replacement (Gibco, 10828-028), 10 μM SP600125, 100 μM cAMP, 20 ng/ml bFGF and penicillin/streptomycin.

LDN193189 IM: DMEM/F12 (Life Technologies, 11330-032), Neurobasal (Life Technologies, 21103-049) (1:1), 0.5% N2 (Invitrogen, 17502048), 1% B27 (Invitrogen, 17504044), 20% KnockOut™Serum Replacement (Gibco, 10828-028), 2.5 μM LDN193189, 100 μM cAMP, 20 ng/ml bFGF and penicillin/streptomycin.

Neuronal MM: DMEM/F12: Neurobasal (1:1), 0.5% N2, 1% B27, 10 μM forskolin, 100 μM cAMP, 20 ng/ml bFGF, 20 ng/ml BDNF, 20 ng/ml GDNF, 20 ng/ml NT3 and penicillin/streptomycin.

NM: DMEM/F12: Neurobasal (1:1), 0.5% N2, 1% B27, 20 ng/ml BDNF, 20 ng/ml GDNF, 20 ng/ml NT3 and penicillin/streptomycin.

### IF staining

IF staining was performed as previously reported 6, 21. Cells were washed three times with PBS, fixed with 4% paraformaldehyde (PFA) for 20 min, and then blocked (in buffer containing 100 mmol/L glycine and 0.3% BSA in PBS) 3 times for 5 min each time. After blocking with 1% BSA for 1.5 h, the primary antibody was prepared, and the cells were incubated with this antibody at 4°C overnight. The next day, the cells were incubated with secondary antibodies for 1.5 h at room temperature, and then a fluorescence microscope was used for imaging and analysis.

The primary antibodies used were against TUJ1 (1:500, Covance, Cat. #MMS435P), MAP2 (1:50, CST, Cat. #4542s), NEUN (1: 100, Millipore, Cat. #MAB377), Choline (1: 100, Abcam, Cat. #ab181023), vGLUT1 (1:500, Synaptic Systems, Cat. #135302), GAD67 (1:50, Abcam, Cat. #ab213508), and TH (1:1000, Abcam, Cat. #ab112), GFAP (1:100, Millipore, Cat. # MAB360), and VIM (1:100, Santa Cruz, Cat. #sc-32322). The secondary antibodies were Alexa Fluor 488 donkey anti-mouse (1:200, Abcam, Cat. #ab150109) and Alexa Fluor 555 donkey anti-rabbit (1: 200, Abcam, Cat. #ab150074) antibodies.

To calculate the positive rate of the cells above, we randomly selected 5-10 fields of view under a fluorescence microscope. Cells positive for TUJ1 or MAP2 with typical neuronal morphology were counted to quantify neurons, Hoechst-positive cells were counted to quantify total cells, and Choline-, vGLUT1-, GAD67- and TH-positive cells were counted to quantify the cells of each subtype. The ratio of the number of Tuj1/Map2-positive cells to the number of Hoechst-positive cells was the positive rate of TUJ1/MAP2. The ratio of the number of choline-/vGLUT1-/GAD67-/TH-positive cells to the number of TUJ1-positive cells was the positive rate of Choline/vGLUT1/GAD67/TH. The above experiments were repeated three times, and the average value was taken for quantitative analysis.

### qRT-PCR

According to the product instructions, TRIzol (Vazyme, Cat. #R401-01) was used to extract total RNA, and a HiScript III RT SuperMix for qPCR kit (Vazyme, Cat. #R323-01) was used to reverse-transcribe the RNA into cDNA. Real-time quantitative PCR was performed on a LongGene Q2000B qPCR instrument using a ChamQ SYBR qPCR Master Mix kit (Vazyme, Cat. #Q711-02) according to the manufacturers' instructions.

### Plasmid construction

Construction of the overexpression vector: First, the circular empty vector pLVX-IRES-ZsGreen1 was linearized by double enzyme digestion, and *CREB1/MAPK8* was connected to the linearized vector by homologous recombination. Subsequently, the plasmid was transformed into *E. coli* for amplification, and colony PCR identification and Sanger sequencing identification were performed.

Construction of the knockout vector: Single guide RNA (sgRNA) was designed according to the gene sequences of *CREB1/MAPK8*. At both ends of the sgRNA, BsmBI enzyme cutting sites were added to generate complementary sticky ends, which were annealed to form double-stranded DNA and then connected to a Lenti-CAS9-sgRNA-puro vector. The ligated product was transformed with competent cells, and after colony PCR verification, positive clones were obtained and sequenced to obtain a lentiviral plasmid expressing sgRNA with the correct sequence.

We thank Beijing Qualityard biotechnology Co., Ltd. for providing the pLVX-IRES-ZsGreen1 plasmid and Genechem Co.,Ltd. for providing the Lenti-CAS9-sgRNA-puro plasmid.

### Lentivirus infection

The above lentiviral recombinant plasmids/vectors (7.5 μg) were cotransfected with VSVG (3 μg) and NRF (4.5 μg) plasmids into 293T cells using Lipofectamine 3000 (Invitrogen) in 60 mm dishes. The virus supernatants were collected 48-72 h after transfection at 37°C under 5% CO_2_. The supernatant was centrifuged (4°C, 2000 rpm, 10 min) and filtered with a 0.45 μm filter, and BJ cells were infected with the supernatant and medium at a ratio of 1:1. After 48 h, fluorescence was observed under a fluorescence microscope, or puromycin screening was performed.

### Electrophysiology

An HD-MEA chip was sterilized in 70% alcohol and then washed three times with sterile deionized water. The chip was placed in a 100 mm petri dish after drying, and a 35 mm dish filled with 2 mL of deionized water was placed in the 100 mm petri dish to provide a humid environment. Next, 400 μL of medium was injected into the chip, which was placed in a 37°C, 5% CO_2_ incubator for pretreatment for 2 days. After 2 days, the culture medium was aspirated, and PDL solution was added to cover the electrode array of the chip. After incubation in the incubator for 1 h, the medium was washed 3 times with deionized water. Cells were seeded on the surface of the treated chip electrode, and various electrophysiology parameters were detected at different time points with MaxOne equipment (MaxWell Biosystems, Switzerland).

Whole-cell recordings of FiNs were performed using a Multiclamp 700B amplifier (Molecular Devices). ACSF, 95% O2 / 5% CO2 blistering was continuously perfused. Pipette solutions contained (in mM) 93 k -gluconate, 16 KCl, 2 MgCl2, 10 HEPES, 4 ATP-Mg, 0.3 GTP-Na2, 10 phosphate, 0.5 Alexa Fluor 568 (Invitrogen), and 0.4% neurobiotin (Invitrogen) (pH 7.25, 290-300 mOsm). Membrane potential was maintained around -70 mV and a step current with an increment of 5 pA was injected to induce an action potential. A step voltage in increments of 10 mV was injected to induce a sodium current. To block the sodium current, TTX was added in the laboratory at a final concentration of 1 µM. To record the spontaneous postsynaptic current, the membrane potential was held around -85 mV. Signals were sampled at 5 kHz with a 2 kHz low-pass filter. Data were analyzed using pClamp 10 software (Clampfit).

### Detection of neurotransmitters

The cell supernatants were collected at the corresponding induction time, centrifuged and filtered to remove dead cells and impurities, and the corresponding neurotransmitters were detected according to the instructions of the ELISA kit (Abcam, ab285238-Dopamine ELISA Kit; ab287792 -Human QuickDetect™ GABA ELISA Kit).

### In vivo transplantation of FiNs

All experiments followed animal welfare policies and were approved by the ethics committee of Guangxi University or The People's Hospital of Guangxi Zhuang Autonomous Region. BJ cells were infected with GFP-tagged lentivirus, and the fluorescence rate was observed under a fluorescence microscope after 48 h (it reached more than 80%). These GFP-BJ cells were induced with the induction method described in this paper and were positive for Tuj1 after 2 days of induction. These induced cells were digested with TryPLE into single cells and resuspended in cold neuronal MM at a density of 5 × 10^4^/μl. The cell suspension was placed on ice and tapped every 5 min. Postnatal 1 day mice (C57BL/6) were immobilized and anesthetized on ice for 5 min. Using a Hamilton syringe (Hamilton, Cat. #701N), 2 μL of the cell suspension was injected into the lateral ventricle of each mouse at a rate of 0.5 μL/min. The same method was used for the contralateral side. The injections were made at sites two-fifths of both eyes of the mice at a depth of 2 mm 6, 44. Then, the mice were sacrificed 7, 30, and 60 days after injection, and their brain tissues were subjected to crytosectioning and IF analysis.

### Cryosectioning and IF analysis

After the mice were sacrificed at the above time points, their brains were removed as soon as possible, placed in liquid nitrogen and quickly frozen into blocks. The samples were precooled in a 4°C refrigerator for 5-10 min to allow the O.C.T. compound to permeate the tissue. The samples were placed in a constant-temperature cryostat for coronal sectioning, placed at room temperature for 30 min, fixed in acetone at 4°C for 5 min, dried in an oven for 20 min, and washed three times with PBS for 5 min each time. Finally, antigen heat retrieval was performed. The sections were air-dried at room temperature for 15 min and incubated with PBS containing 10% donkey serum for 1 h at room temperature. A primary antibody against TUJ1/MAP2 was diluted at a concentration of 1:50, and the sections were incubated with this antibody at room temperature for 2 h. The secondary antibody, anti-mouse-555/anti-rabbit-555, was diluted at a concentration of 1:200. The sections were incubated with the secondary antibody at 37°C for 1 h and washed with PBS 3 times for 5 min each time. Hoechst was added dropwise to stain the nuclei, and the sections were incubated at room temperature for 15 min. Then, 10 μL of neutral gum was added dropwise to seal the slides, and the slides were placed under a fluorescence microscope for observation and imaging.

### Induced cell dynamics tracking

The cells were inoculated in a 96-well plate, an appropriate amount of PBS was added to the wells around the inoculated wells to maintain a suitable humid environment. The abovementioned 96-well plates were placed in a BioTek Cytation5 plate, and the appropriate exposure and length of exposure were set. Shots were taken every 2 h for a total of 132 h of tracking, and the induced cells were cultivated in accordance with the above steps.

### ScRNA-seq library construction and sequencing

ScRNA-seq was performed on BJ cells and D3 cell samples (3 days post-induction) using a 10× Genomics system. Briefly, dissociated cells (~10,000 cells per sample) were loaded into a 10× Genomics Chromium Single Cell system using Chromium Single Cell 3' Reagent Kits v3.1 (10× Genomics, Pleasanton, CA). ScRNA libraries were generated by following the manufacturer's instructions. The libraries were pooled and sequenced on an Illumina NovaSeq 6000. The sequencing reads were processed through the Cell Ranger 4.0.0 pipeline (10× Genomics) using the default parameters.

### ScATAC-seq library construction and sequencing

ScATAC-seq was performed on BJ cells and D3 cell samples (3 days post-induction) using a 10× Genomic Single Cell ATAC Reagent v1.1 Kit following the manufacturer's instructions. The libraries were pooled and sequenced on an Illumina NovaSeq 6000. The sequencing data were processed through the Cell Ranger ATAC 1.1.0 pipeline (10x Genomics) using the default parameters.

### Bulk RNA-seq (mRNA-seq) library construction and sequencing

Total RNA was extracted from cells using TRIzol® Reagent according the manufacturer's instructions (Invitrogen), and genomic DNA was removed using DNase I (TaKaRa). The RNA-seq transcriptome library was prepared following the TruSeq^TM^ RNA Sample Preparation Kit from Illumina using 1 μg of total RNA. After quantification, the RNA-seq sequencing library was sequenced with the Illumina NovaSeq 6000 sequencer in paired-end mode (2 × 150 bp read length). Then, the clean reads were separately aligned to the reference genome in orientation mode using HISAT2 software (http://ccb.jhu.edu/software/hisat2/index.shtml). The mapped reads of each sample were assembled with StringTie (https://ccb.jhu.edu/software/stringtie/index.shtml? t=example) in a reference-based approach.

### ScRNA-seq analysis

The clean scRNA-seq reads for all of the samples were mapped to the human reference genome hg38 using Cell Ranger v.4.0.0 45. The expression matrices were loaded into R v.4.1.0 using the function Read10× in Seurat (v.4.1.0) 46 and then merged together by column. This resulted in a total of 11,488 cells from samples at 3 days post-induction and 17,504 cells from BJ cells. Cell-level quality control was performed by filtering the cells by (1) total UMI counts of no more than 5,000 but higher than 500; (2) gene numbers higher than 500 but less than 2500; and (3) mitochondrial gene percentages less than 10. The expression level of each gene in each cell was normalized using the function NormalizeData with the default parameters. Cluster-level quality control was performed after the standard Seurat clustering pipeline was implemented using the following functions in order: FindVariableFeatures with all features, ScaleData, RunPCA, FindNeighbors with the first 16 principal components (PCs) and FindClusters with resolution 0.2, otherwise default settings. Clusters with fewer than 50 cells were removed. After quality control, 10575 cells from cells at 3 days post-induction and 2,461 cells from BJ samples remained.

Genes that were differentially expressed between clusters (cluster markers) were identified with the FindAllMarkers function using a Wilcoxon rank sum test and a minimum upregulation of 0.05 log-fold. GO analysis of all gene groups was performed using the function enrichGO in the R package clusterProfiler 47.

### Construction of a trajectory using DEGs

Monocle 2 ordering was conducted by using the set of variable genes with the default parameters, except that we specified reduction_method = “DDRTree” in the reduceDimension function 48. The key regulator factor was submitted to the STRING database to infer regulatory networks based on known interaction relationships (supported by data from curated databases, experiments, and text mining). Factors without any interactions with other proteins were removed from the network. The network was visualized with Cytoscape (v3.9.0).

### ScATAC-seq analysis

All the analyses (UMAP dimension reduction, cluster identification, and identification of differentially accessible regions) were performed according to the Signac (v1.6.0) 49 vignettes, and the default parameter settings were used to construct cell trajectories with Monocle 3 50.

### Bulk mRNA-seq analysis

The data were analyzed on the Majorbio Cloud Platform (https://cloud.majorbio.com/).

### Quantification and statistical analysis

Statistical analysis of quantified data was performed using GraphPad software. Significance was calculated with Student's t test or one-way ANOVA, unless otherwise stated. The data are presented as the mean±SEM. *p < 0.05, **p < 0.01, ***p < 0.001.

## Supplementary Material

Supplementary figures and key resources table.

Supplementary video for cell tracking.
