# Supplementary material for: Forskolin-driven conversion of human somatic cells into induced neurons through regulation of the cAMP-CREB1-JNK signaling
Source: Theranostics. 2024 Feb 11;14(4):1701–19. doi: 10.7150/thno.92700 (PMC10879881; doi:10.7150/thno.92700)
Supplement: Supplementary file 1 — Supplementary figures and key resources table. [file thnov14p1701s1.pdf]

KEY RESOURCES TABLE

REAGENT or RESOURCE	SOURCE	IDENTIFIER
Antibodies		
Mouse monoclonal anti-TUJ1	Santa Cruz	Cat#sc-5274; RRID: AB_2288090
Rabbit polyclonal anti-MAP2	CST	Cat#4542s; RRID: AB_10693782
Mouse monoclonal anti-NEUN	Millipore	Cat#MAB377; RRID: AB_2298772
Rabbit monoclonal anti-Choline	Abcam	Cat#ab181023; RRID: AB_2687983
Rabbit polyclonal anti-vGLUT1	Synaptic Systems	Cat#135302; RRID: AB_887877
Rabbit monoclonal anti-GAD67	Abcam	Cat#ab213508
Rabbit monoclonal anti-TH	Abcam	Cat#ab137869; RRID: AB_2801410
Mouse monoclonal anti-GFAP	Millipore	Cat#MAB360; RRID: AB_11212597
Mouse monoclonal anti-Vimentin	Santa Cruz	Cat#sc-32322; RRID: AB_628436
Alexa Fluor 488 Donkey Anti-mouse	Abcam	Cat#ab150109; RRID: AB_2571721
Alexa Fluor 555 Donkey Anti-rabbit	Abcam	Cat#ab150074; RRID: AB_2636997
Chemicals, Peptides, and Recombinant Proteins		
Forskolin	Selleck	S2449; CAS: 66575-29-9
cAMP	Sigma-Aldrich	20-198
8-Bromo-cAMP	Selleck	S7857; CAS: 76939-46-3
SP600125	Selleck	S1460; CAS: 129-56-6
LDN-193189	Selleck	S2618; CAS: 1062368-24-4
Recombinant Human FGF-basic (bFGF)	Peprotech	Cat#100-18B
Recombinant Human BDNF	Peprotech	Cat#450-02
Recombinant Human GDNF	Peprotech	Cat#450-10
Recombinant Human NT-3	Peprotech	Cat#450-03
Critical Commercial Assays		
RNA isolater Total RNA Extraction Reagent	Vazyme	Cat#R401-01
HiScript III RT SuperMix for qPCR kit	Vazyme	Cat#R323-01
ChamQ SYBR qPCR Master Mix kit	Vazyme	Cat. #Q711-02
Deposited Data		
RNA-seq data	This paper	GEO: GSE206514
ScRNA-seq data	This paper	GEO: GSE206766

ScATAC-seq data	This paper	GEO: GSE206767
Experimental Models: Cell Lines		
BJ cells	ATCC	CRL-2522
Oligonucleotides		
Primer: GAPDH Forward: TCCTGCACCACCAACTGCTT	This paper	N/A
Primer: GAPDH Reverse: ACCATGAGTCCTTCCACGAT	This paper	N/A
Primer: TUJ1 Forward: GCAACTACGTGGGCGACT	This paper	N/A
Primer: TUJ1 Reverse: CGAGGCACGTACTTGTGAGA	This paper	N/A
Primer: DCX Forward: CAAGTCTAAGCAGTCTCCCATC	This paper	N/A
Primer: DCX Reverse: ATAGCCCTGTTGGACACTTG	This paper	N/A
Primer: MAP2 Forward: CAGGAGACAGAGATGAGAATTCC	This paper	N/A
Primer: MAP2 Reverse: CAGGAGTGATGGCAGTAGAC	This paper	N/A
Primer: SYN1 Forward: CCCCAATCACAAAGAAATGCTC	This paper	N/A
Primer: SYN1 Reverse: ATGTCCTGGAAGTCATGCTG	This paper	N/A
Primer: COL1A1 Forward: GAGGGCCAAGACGAAGACATC	This paper	N/A
Primer: COL1A1 Reverse: CAGATCACGTCATCGCACAAC	This paper	N/A
Primer: CTGF Forward: CATCTCCACCCGGGTTACCAA	This paper	N/A
Primer: CTGF Reverse: AGTACGGATGCACTTTTTGC	This paper	N/A
Primer: THY1 Forward: ATCGCTCTCCTGCTAACAGTC	This paper	N/A
Primer: THY1 Reverse: CTCGTACTGGATGGGTGAACT	This paper	N/A
Primer: DKK3 Forward: CTGGGAGCTAGAGCCTGATG	This paper	N/A
Primer: DKK3 Reverse: TCATACTCATCGGGGACCTC	This paper	N/A
Primer: CREB1 Forward: TGTAACTGATTCCCAAAAGCG	This paper	N/A
Primer: CREB1 Reverse: TCTTCTTCAATCCTTGGCACT	This paper	N/A
Primer: MAPK8 Forward: GACGACGCGGCTTGGATT	This paper	N/A
Primer: MAPK8 Reverse: TAAGGCTGCAAGACCGGC	This paper	N/A
Software and Algorithms		

GraphPad Prism 9	GraphPad Software	https://www.graphpad.com/
ImageJ	NIH	https://imagej.nih.gov/ij/download.html
R version 4.0.5	The R Foundation for Statistical Computing	https://www.r-project.org/

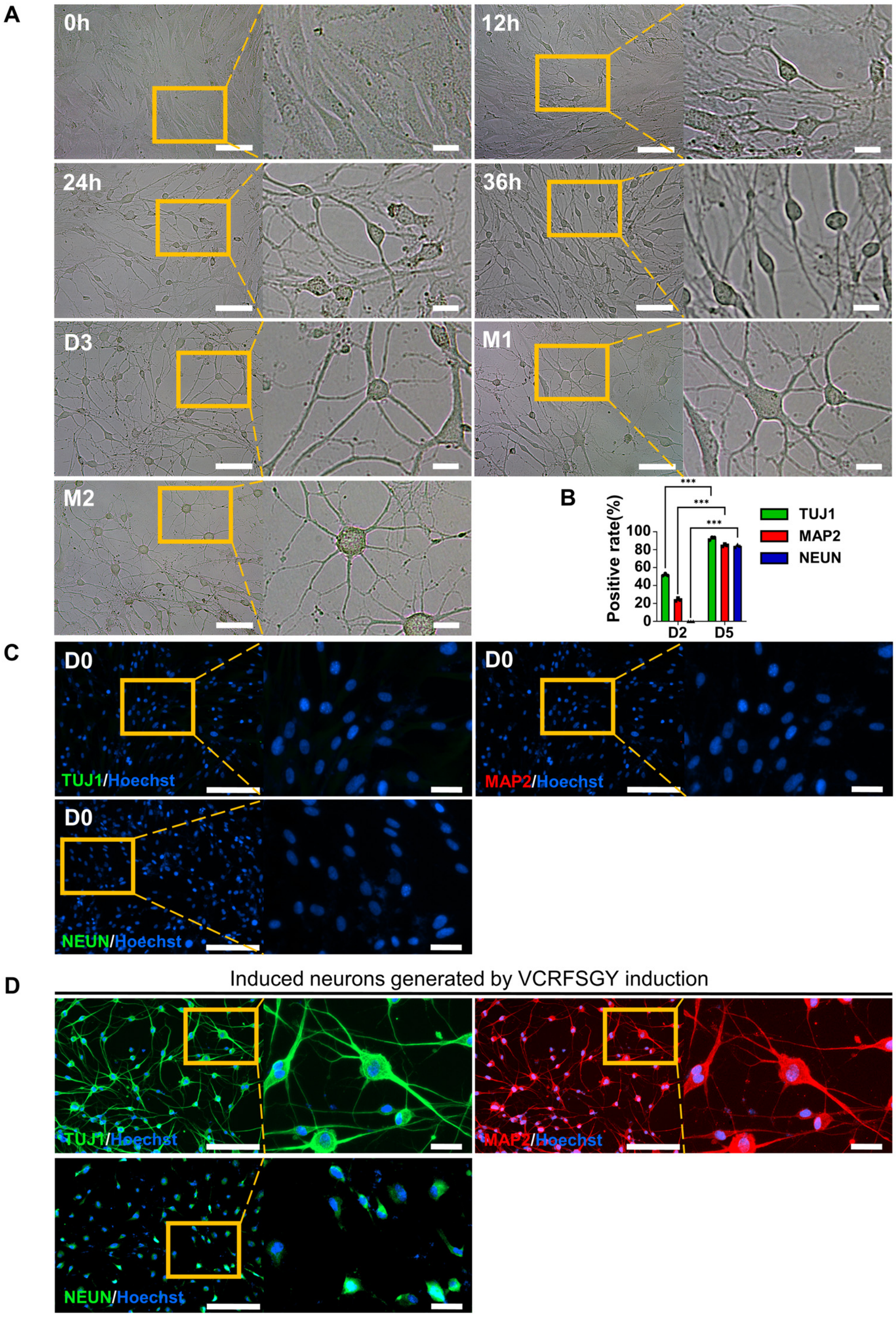
A
0h
12h
24h
36h
D3
M1
M2
B
Positive rate(%)
100
80
60
40
20
0
D2
D5
***
***
***
TUJ1
MAP2
NEUN
C
D0
TUJ1/Hoechst
D0
MAP2/Hoechst
D0
NEUN/Hoechst
D
Induced neurons generated by VCRFSGY induction
TUJ1/Hoechst
MAP2/Hoechst
NEUN/Hoechst

Figure S1. Morphological changes in induced cells from the BJ cell stage to two months post-induction and immunofluorescence staining for TUJ1, MAP2, and NEUN.

A. Brightfield and regional magnification of forskolin (F) induction at 0, 12, 24 and 36 h; 1 month (M1); and 2 months (M2). Scale bars, 100 μm. Magnification scale bars, 20 μm.

B. Statistical chart of the changes in TUJ1-, MAP2-, and NEUN-positive rates in cells induced with forskolin for 2 days and 5 days (mean ± SEM, n=3 biological replicates, ***$p < 0.001$, one-way ANOVA).

C. BJ cells (day 0) stained negative for the neuronal markers TUJ1, MAP2 and NEUN and were used as negative controls. Scale bars, 200 μm. Magnification scale bars, 40 μm.

D. Human induced neuronal cells generated from somatic cells by induction with a small molecule cocktail (VCRFSGY) as described in a previous report stained positively for TUJ1, MAP2 and NEUN and were used as positive controls. Scale bars, 200 μm. Magnification scale bars, 40 μm.

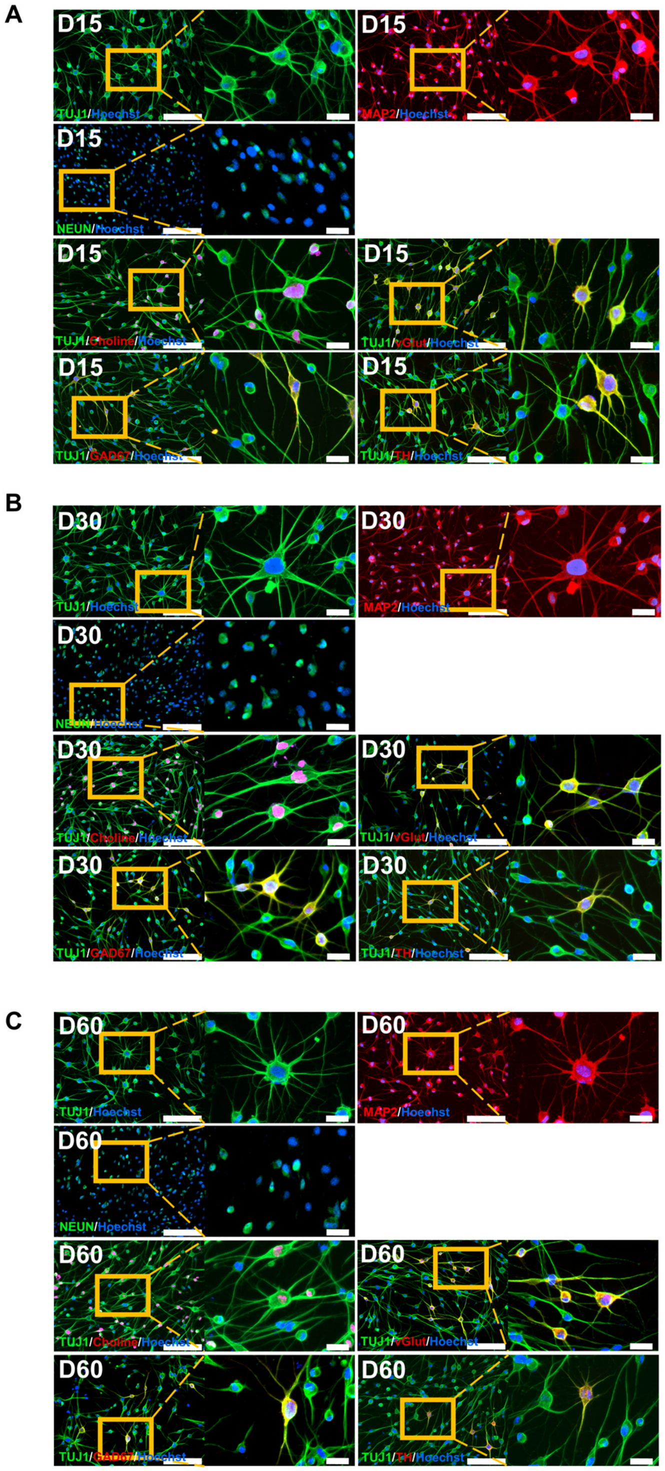

Figure S2. Immunofluorescence staining of FiNs at D15, -30, -60.

A-C. Immunofluorescence staining for neuronal markers, TUJ1 (green), MAP2 (red), NEUN (green), choline (red), vGlut (red), GAD67 (red), TH (red) and Hoechst (blue) at D15, -30, and -60. TUJ1-positive cells are shown in green. Scale bars, 200 μm. Magnification scale bars, 40 μm.

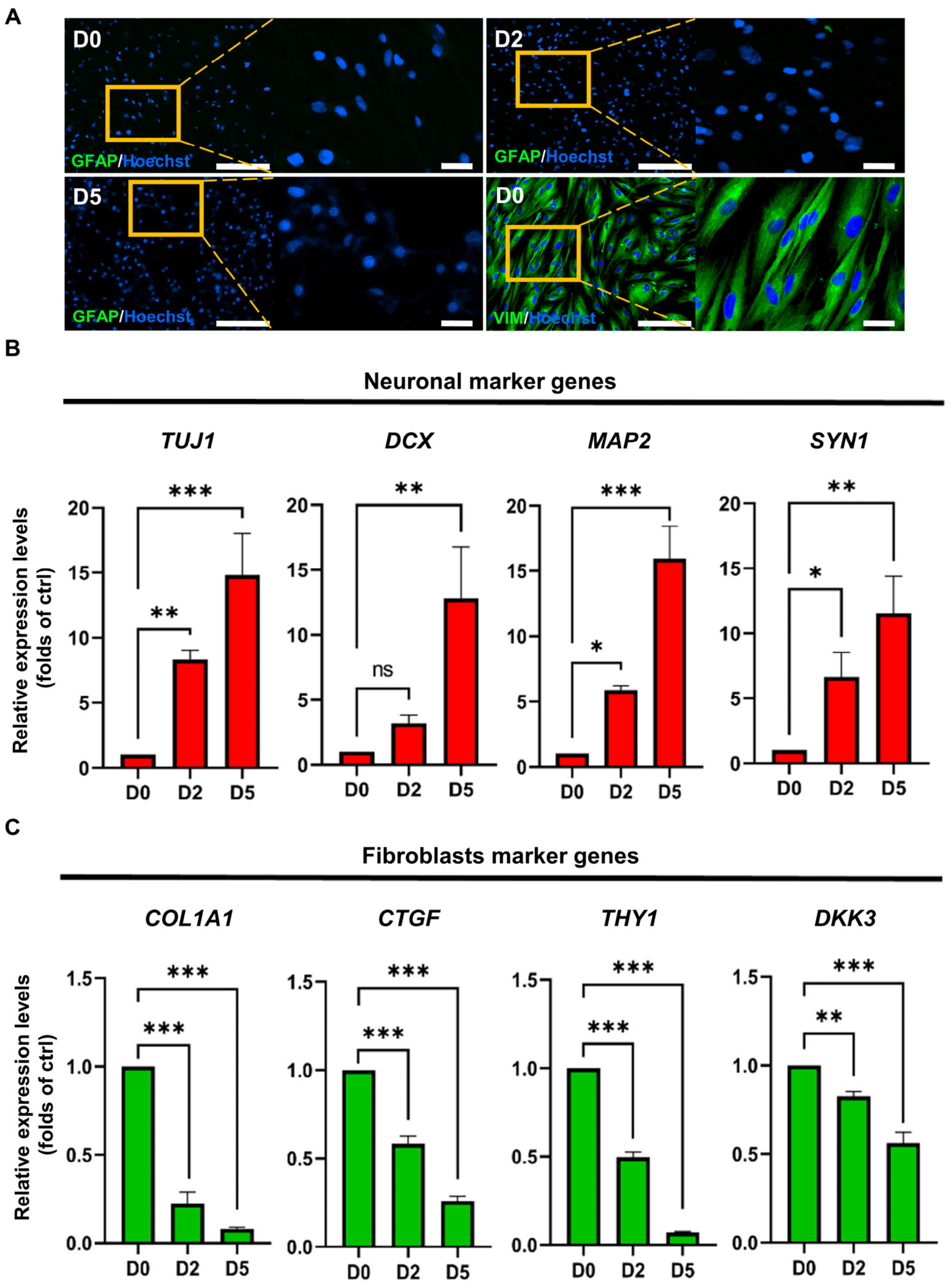
A
D0
GFAP/Hoechst
D2
GFAP/Hoechst
D5
GFAP/Hoechst
D0
VIM/Hoechst
B
Neuronal marker genes
TUJ1
DCX
MAP2
SYN1
Relative expression levels (folds of ctrl)
D0
D2
D5
ns
C
Fibroblasts marker genes
COL1A1
CTGF
THY1
DKK3
Relative expression levels (folds of ctrl)

Figure S3. I**mmunofluorescence staining for GFAP and VIM, qRT–PCR for the expression of marker genes.**

A. Cells stained negative for an astrocyte marker (GFAP) at day 0 (D0), day 2 (D2) and day 5 (D5) during forskolin induction. BJ cells (D0) stained positive for a fibroblast marker (Vimentin/VIM). Scale bars, 200 µm. Magnification scale bars, 40 µm.

B-C. qRT–PCR analysis of cells after forskolin induction for 0, 2, and 5 days (mean ± SEM, n=3 biological replicates, $*p < 0.05$, $**p < 0.01$, $***p < 0.001$, one-way ANOVA).

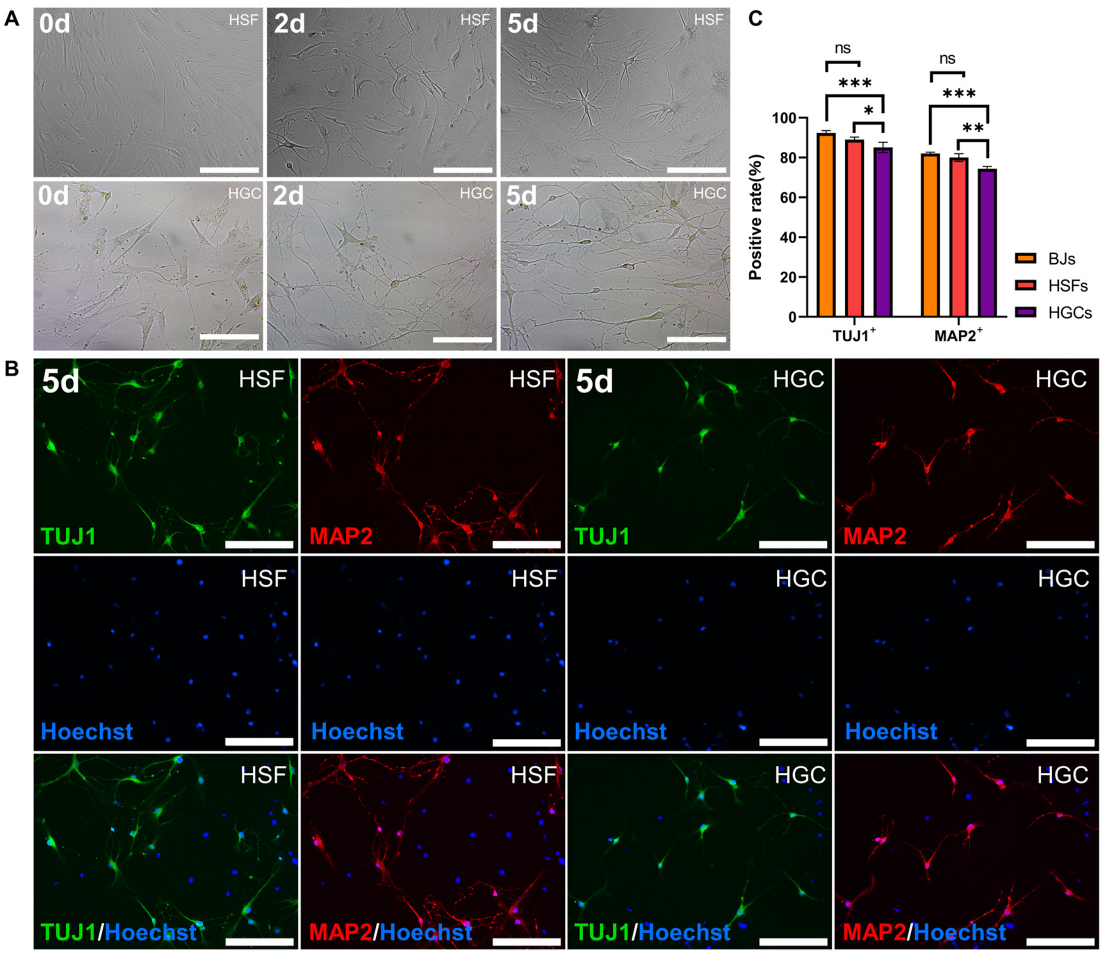

Figure S4. Human adult somatic cells (HSFs and HGCs) were conversed into induced neurons under forskolin induction.

A. Morphological changes in human adult skin fibroblasts (HSFs, age 24, male) and human adult ovarian granule cells (HGCs, age 35, female) induced by forskolin at 0-, 2-, and 5 days post induction. Scale bars, 200 μm.

B. TUJ1 (green) and MAP2 (red) immunostaining (co-staining) of induced neurons (iNs) generated from HSFs and HGCs induced by forskolin at 5 days post induction. Scale bars, 200 μm.

C. The positive rate of TUJ1 and MAP2 immunofluorescence of iNs derived from BJs, HSFs and HGCs induced by forskolin at 5 days post induction (mean±SEM, n=3 biological replicates, *p < 0.05, **p < 0.01, ***p < 0.001, two-way ANOVA).

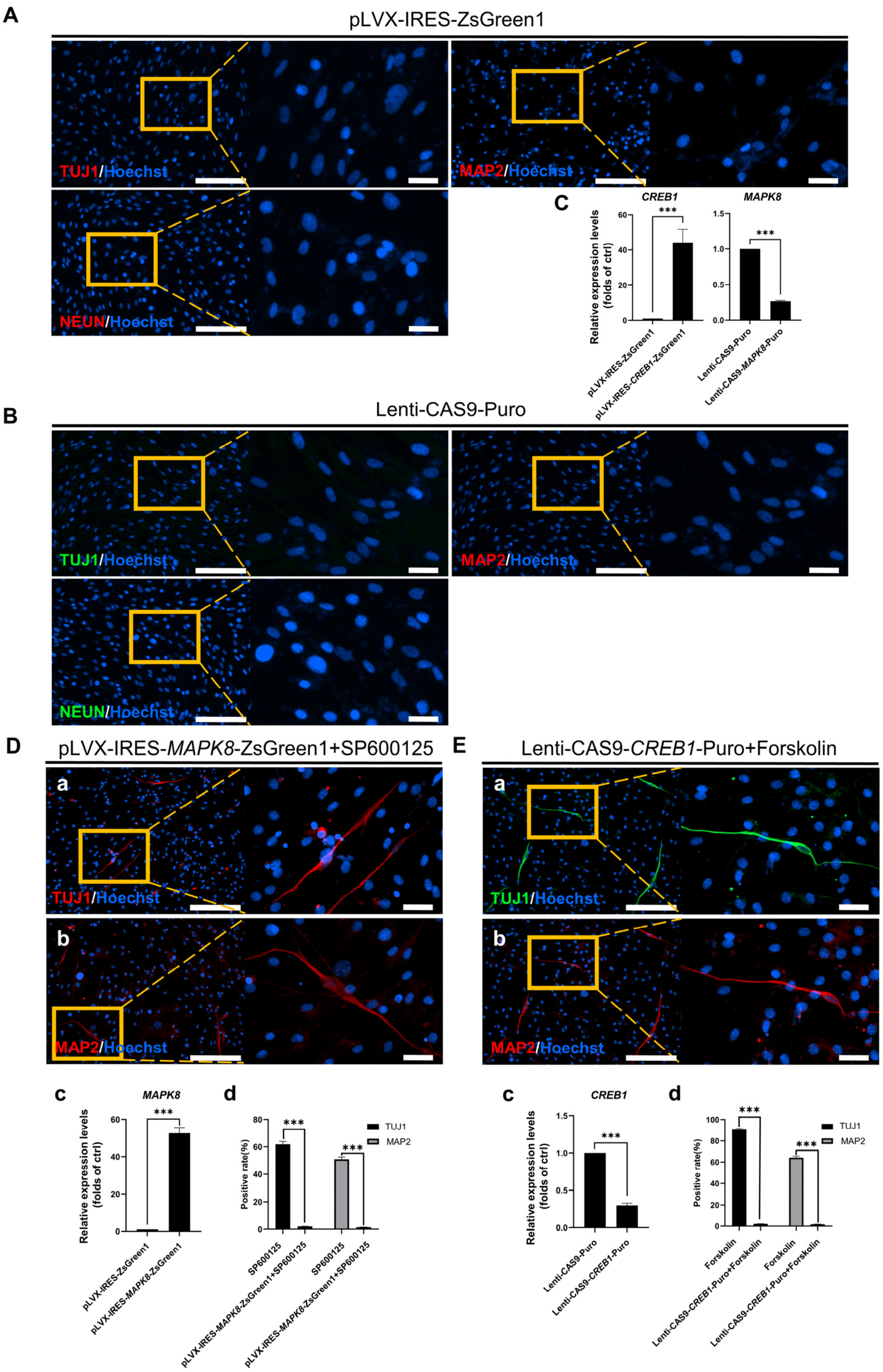
A
pLVX-IRES-ZsGreen1
TUJ1/Hoechst
MAP2/Hoechst
NEUN/Hoechst
C
CREB1
MAPK8
Relative expression levels (folds of ctrl)
***
pLVX-IRES-ZsGreen1
pLVX-IRES-CREB1-ZsGreen1
Lenti-CAS9-Puro
Lenti-CAS9-MAPK8-Puro
B
Lenti-CAS9-Puro
TUJ1/Hoechst
MAP2/Hoechst
NEUN/Hoechst
D
pLVX-IRES-MAPK8-ZsGreen1+SP600125
a
TUJ1/Hoechst
b
MAP2/Hoechst
E
Lenti-CAS9-CREB1-Puro+Forskolin
a
TUJ1/Hoechst
b
MAP2/Hoechst
c
MAPK8
Relative expression levels (folds of ctrl)
***
pLVX-IRES-ZsGreen1
pLVX-IRES-MAPK8-ZsGreen1
d
Positive rate(%)
TUJ1
MAP2
***
SP600125
pLVX-IRES-MAPK8-ZsGreen1+SP600125
c
CREB1
Relative expression levels (folds of ctrl)
***
Lenti-CAS9-Puro
Lenti-CAS9-CREB1-Puro
d
Positive rate(%)
TUJ1
MAP2
***
Forskolin
Lenti-CAS9-CREB1-Puro+Forskolin

Figure S5. BJ cells transfected with pLVX-IRES-ZsGreen1 or Lenti-CAS9-Puro were negative for TUJ1, MAP2, NEUN, and *JNK (MAPK8)* overexpression or *CREB1* downregulation, which significantly interrupted SP600125 or forskolin induction, respectively.

A. BJ cells transfected with pLVX-IRES-ZsGreen1 (empty vector) stained negative for the neuronal markers TUJ1 (red), MAP2 (red), and NEUN (red) and were used as negative controls. Scale bars, 200 μm. Magnification scale bars, 40 μm.

B. BJ cells transfected with Lenti-CAS9-Puro (empty vector) stained negative for the neuronal markers TUJ1 (green), MAP2 (red), and NEUN (green) and were used as negative controls. Scale bars, 200 μm. Magnification scale bars, 40 μm.

C. The qRT–PCR results confirmed *CREB1* overexpression or *JNK* (*MAPK8)* downregulation in the cells transfected with pLVX-IRES-*CREB1*-ZsGreen1 or Lenti-CAS9-*MAPK8*-Puro, respectively (mean ± SEM, n = 3 biological replicates, ***p < 0.001).

D. *JNK (MAPK8)* overexpression significantly interrupted the effects of SP600125 on induced neuron generation. a-b, Immunofluorescence staining for TUJ1 and MAP2. c, qRT–PCR data demonstrated that *JNK* was upregulated in the cells transfected with pLVX-IRES-*MAPK8*-ZsGreen1. d, *JNK* overexpression decreased the rate of TUJ- and MAP2 positive cells under SP600125 induction. Scale bars, 200 μm. Magnification scale bars, 40 μm (means ± SEMs, n = 3 biological replicates, ***p < 0.001).

E. *CREB1* downregulation significantly interrupted the effects of forskolin on neuronal generation. a-b, Immunofluorescence staining for TUJ1 and MAP2. c, qPCR data demonstrated that *CREB1* was downregulated in the cells transfected with Lenti-CAS9-*CREB1*-Puro. d, *CREB1* downregulation decreased the rates of TUJ- and MAP2-positive cells under forskolin induction. Scale bars, 200 μm. Magnification scale bars, 40 μm (means ± SEMs, n = 3 biological replicates, ***p < 0.001).

A
B
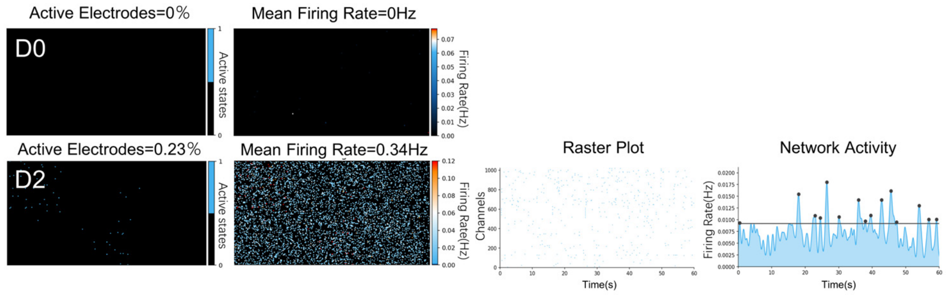

C

Days	Dopamine (DA) ($ng \cdot mL^{-1}$)	GABA ($pg \cdot mL^{-1}$)
0d	0	0
5d	5.1±0.93	63±2.5

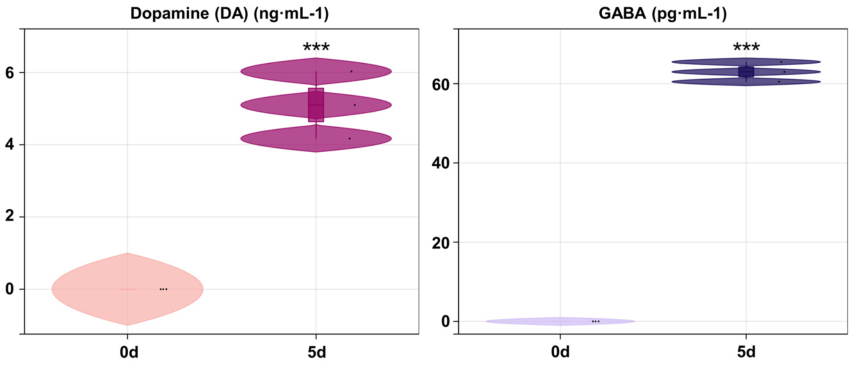

Figure S6. Electrophysiological properties of BJ cells (D0), induced cells (D2) and the neurotransmitter examination of induced neurons.

A. HD-MEA electrical images showing the 2D spatial distributions of active electrodes (%) and the mean firing rate (Hz) on day 0 (D0/BJ cells). No significant signals were detected for these negative controls (n = 3 biological replicates).

B. HD-MEA electrical images showing the spatial distributions of active electrodes (%) and the mean firing rate (Hz) on day 2 (D2). On D2, the HD-MEA chip detected the electrical signals (0.23%) of cells in each channel and the network activity (firing rate (Hz)) (0.34 Hz) with a cycle of 60 seconds (n=3 biological replicates).

C. Dopamine (DA) and GABA neurotransmitters secreted by induced cells at 0-, 5 days post forskolin induction, respectively (Unpaired t test. Two-tailed, n=3 biological replicates, ***$p < 0.001$).

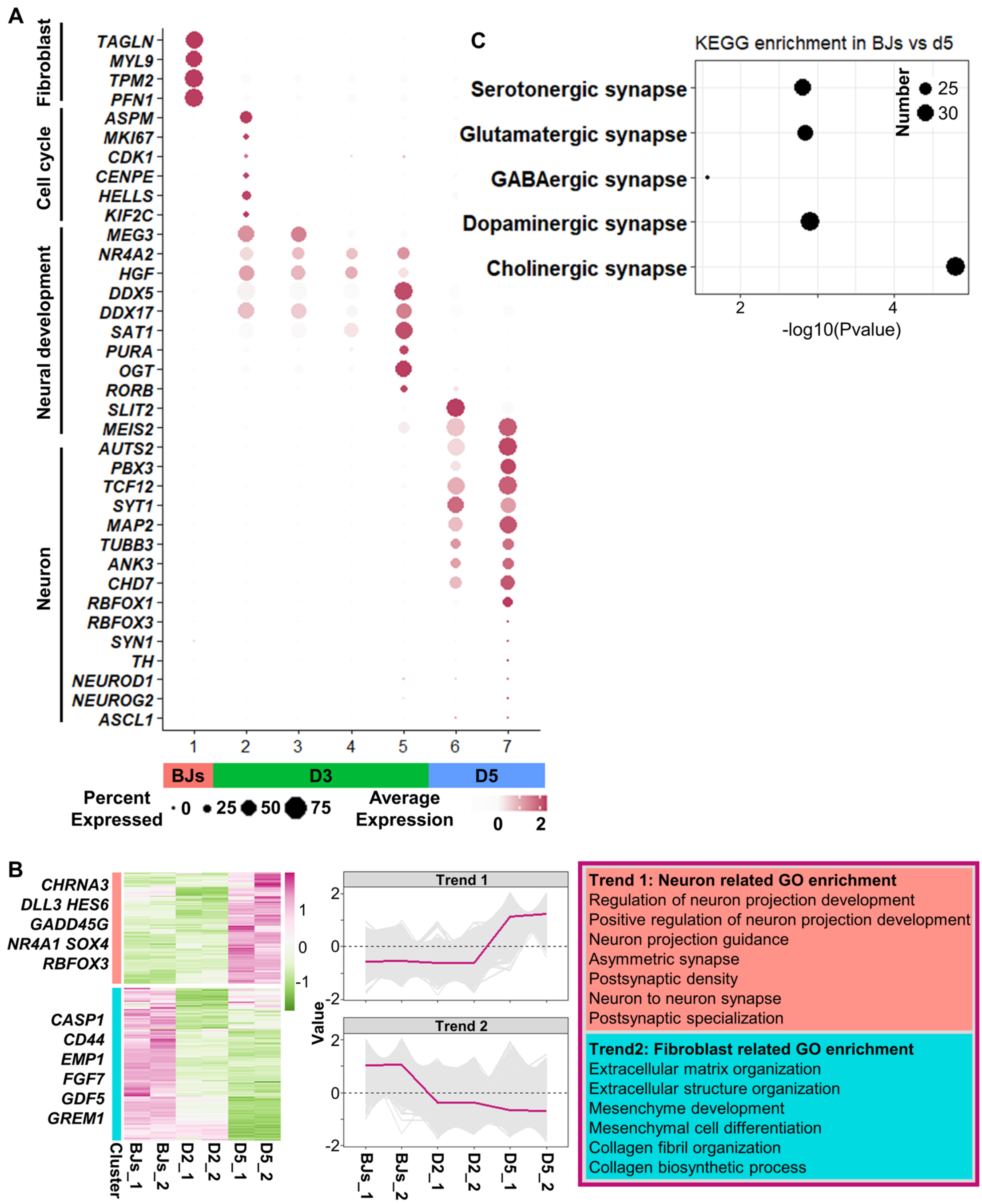

Figure S7. Bulk mRNA-seq and scRNA-seq analysis revealed the conversion of BJ cells into FiNs.

A. Dot pot of scRNA-seq data showing the expression pattern of fibroblast-, cell cycle-, neural development- and neuron-related genes in the putative trajectory.

B. Heatmap and GO enrichment of differentially expressed genes (DEGs) from the mRNA-seq data (BJ cells, days 2 and 5 (D2 and D5)). The DEGs were divided into two clusters based on their expression patterns (Trends 1 and 2). The DEGs of Trend 1 were enriched with fibroblast-related GO terms, and the DEGs of trend 2 were enriched with neuron-related GO terms.

C. KEGG enrichment analysis showed that the neuronal subtype terms were enriched in D5 cells samples.

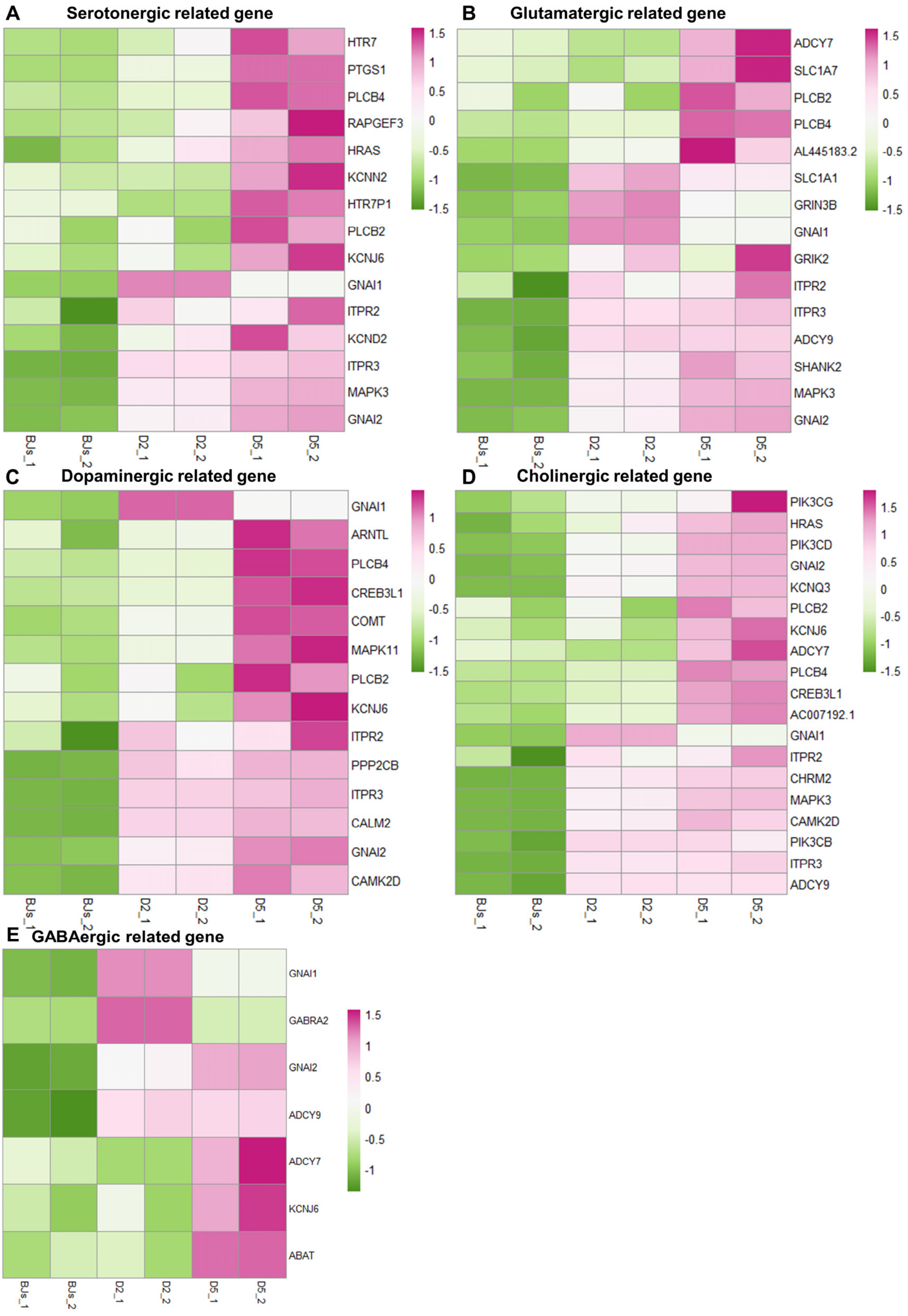

Figure S8. Heatmap shows significant upregulation of neuronal subtype genes.

A-E. Heatmap of Serotonergic related genes, Glutamatergic related genes, Dopaminergic related gene, Cholinergic related gene and GABAergic related gene showing significant upregulation during the reprogramming process.

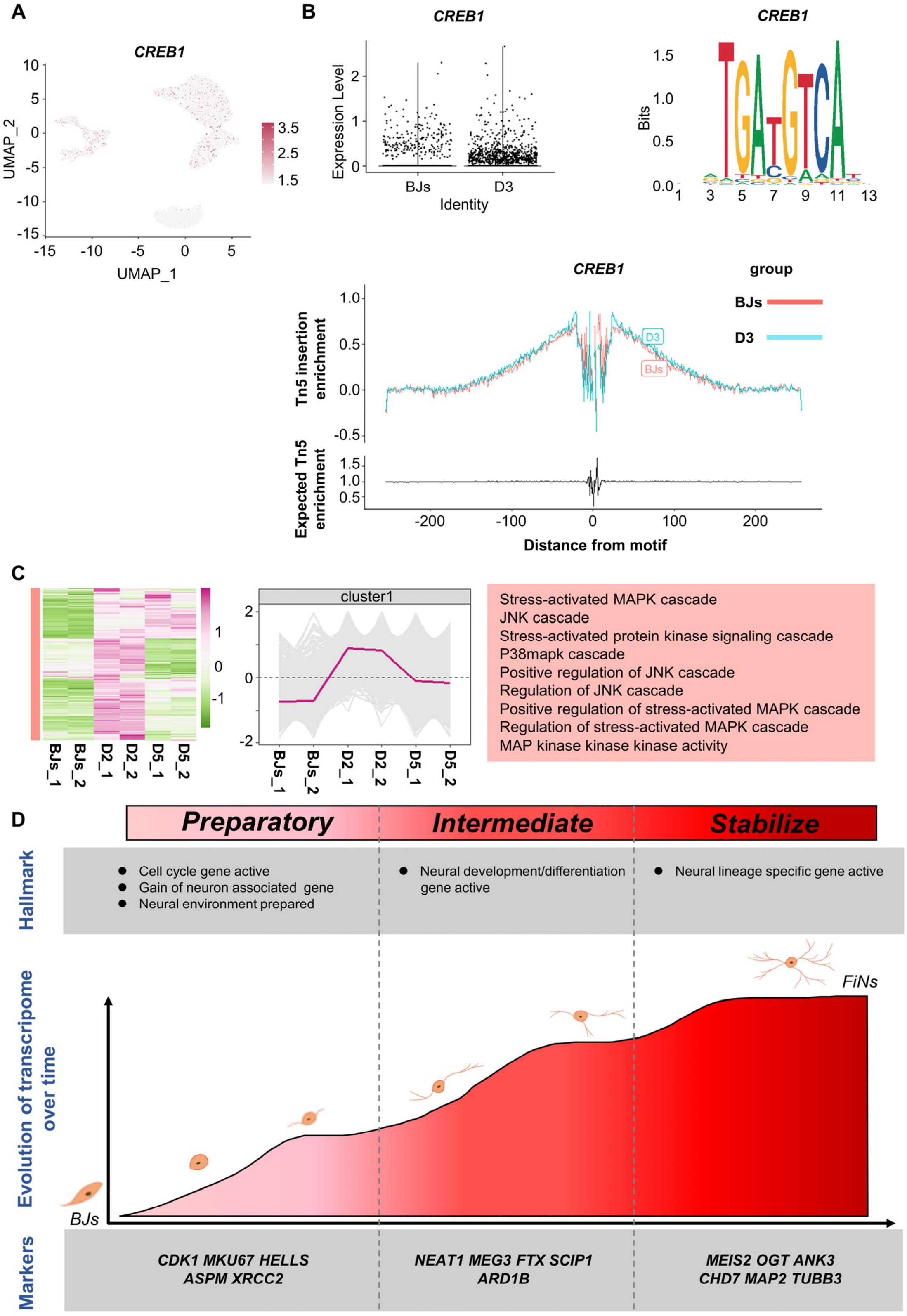

A
CREB1
UMAP_2
UMAP_1
B
CREB1
Expression Level
BJs
D3
Identity
CREB1
Bits
CREB1
group
BJs
D3
Tn5 insertion enrichment
Expected Tn5 enrichment
Distance from motif
C
cluster1
BJs_1
BJs_2
D2_1
D2_2
D5_1
D5_2
Stress-activated MAPK cascade
JNK cascade
Stress-activated protein kinase signaling cascade
P38mapk cascade
Positive regulation of JNK cascade
Regulation of JNK cascade
Positive regulation of stress-activated MAPK cascade
Regulation of stress-activated MAPK cascade
MAP kinase kinase kinase activity
D
Preparatory
Intermediate
Stabilize
Hallmark
Cell cycle gene active
Gain of neuron associated gene
Neural environment prepared
Neural development/differentiation gene active
Neural lineage specific gene active
Evolution of transcripome over time
BJs
FiNs
Markers
CDK1 MKU67 HELLS ASPM XRCC2
NEAT1 MEG3 FTX SCIP1 ARD1B
MEIS2 OGT ANK3 CHD7 MAP2 TUBB3

Figure S9. Single-cell sequencing (scRNA-seq and scATAC-seq) analysis revealed that *CREB1* and *JNK* play important initial roles in FiN reprogramming.

A. UMAP plot of *CREB1* expression in different clusters from the scRNA-seq data. *CREB1* is expressed under forskolin induction during the FiN reprogramming process.

B. The violin plot (left), the transcription factor (TF) footprints (right) of *CREB1* motifs, and the *CREB1 Tn5* insertion bias track (below) showed that *CREB1* was significantly enriched in the D3 cells sample in the scATAC-seq data.

C. Heatmap of DEGs in the bulk mRNA-seq data (BJ cells, day-2 and day-5 samples). The trend line is in the middle, and the GO categories are shown on the right. The results indicate that the MAPK/JNK pathway is closely involved in the early stage of FiN reprogramming.

D. Cell fate conversion model for the FiN reprogramming process. Three major states (preparatory, intermediate, and stabilized) make up the reprogramming process induced by forskolin. The schematic diagram of cell morphological changes beside the black line indicates the morphological changes that occur during each reprogramming state. Each stage's genes are presented at the bottom. The characteristics of each stage are described at the top.
